# Real‐Time In Vivo Visualization of Tumor‐Associated Macrophage Reprogramming Using a Nitric Oxide‐Activatable NIR‐II Nanoinducer

**DOI:** 10.1002/advs.202524367

**Published:** 2026-03-10

**Authors:** Qian Chen, Meng Li, Tuanwei Li, Chen Yang, Xiaohu Yang, Hongchao Yang, Yejun Zhang, Chunyan Li, Qiangbin Wang

**Affiliations:** ^1^ CAS Key Laboratory of Nano‐Bio Interface, Suzhou Key Laboratory of Functional Molecular Imaging Technology, Division of Nanobiomedicine and i‐Lab Suzhou Institute of Nano‐Tech and Nano‐Bionics Chinese Academy of Sciences Suzhou China; ^2^ Medical Science and Technology Innovation Center, The Affiliated Suzhou Hospital of Nanjing Medical University, Suzhou Municipal Hospital Gusu School of Nanjing Medical University Suzhou China

**Keywords:** NIR‐II window, ratiometric imaging, tumor‐associated macrophage reprogramming, tumor microenvironment

## Abstract

Tumor‐associated macrophages (TAMs) are key regulators of the tumor microenvironment (TME). They typically adopt an M2‐like phenotype that promotes tumor progression by providing survival signals, suppressing anti‐tumor immunity, and facilitating pre‐metastatic niche formation. Reprogramming TAMs toward an anti‐tumor phenotype has emerged as a promising therapeutic strategy, with the repolarization of M2‐like TAMs into an M1‐like phenotype being central to this approach. Here, a nitric oxide (NO)‐activatable near‐infrared‐II (NIR‐II) fluorescence/photoacoustic nanoinducer (I/E@M2pep) that selectively targets M2‐like TAMs and reprograms them toward an M1‐like phenotype, thereby enhancing anti‐tumor efficacy is reported. In this construct, the M2pep peptide enables M2‐like TAM targeting, IPI549 reprograms them toward an M1‐like phenotype while inducing NO production, and the NO‐activatable NIR‐II probe (ETNO) allows for in vivo visualization of macrophage repolarization via NIR‐II fluorescence/photoacoustic imaging. In a mouse breast cancer model, intravenous administration of I/E@M2pep produced a ratiometric NIR‐II photoacoustic signal change that correlated with M2‐to‐M1 repolarization. Furthermore, combining this nanoinducer with a CD47 monoclonal antibody markedly enhanced anti‐tumor immunity through M1 macrophage‐mediated tumor killing and TME remodeling. This work presents an effective in vivo strategy that simultaneously facilitates and visualizes TAM repolarization, holding promise for broader applications in studying tumor initiation, metastasis, and treatment response.

## Graphical abstract

Reprogramming tumor‐associated macrophages is a promising therapeutic strategy for solid tumors. Here, a nitric oxide (NO)‐activatable NIR‐II fluorescence/photoacoustic nanoinducer (I/E@M2pep) that simultaneously facilitates and visualizes the repolarization of TAMs to an M1‐like phenotype is reported, thereby enhancing anti‐tumor efficacy through M1 macrophage‐mediated tumor killing and the tumor microenvironment remodeling.

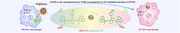

## 1 Introduction

Tumor‐associated macrophages (TAMs) represent one of the most abundant immune cell populations within the tumor microenvironment (TME) and play central roles across tumor initiation, progression, and metastasis [1, 2, 3, 4]. TAMs are typically skewed toward an M2‐like, pro‐tumor phenotype, characterized by the secretion of growth and pro‐angiogenic factors, extracellular matrix (ECM) remodeling, and the contribution to an immunosuppressive TME through cytokines such as IL‐10 and TGF‐β [5, 6, 7, 8, 9, 10, 11, 12, 13, 14, 15, 16]. In contrast, M1‐like macrophages mediate tumor cell killing by releasing cytotoxic mediators like nitric oxide (NO) and reactive oxygen species (ROS). Also, they can exert tumor phagocytosis and promote antigen presentation to foster anti‑tumor immunity [17, 18, 19]. Consequently, immunotherapeutic strategies that reprogram M2 TAMs toward an M1‑like phenotype have emerged as attractive therapeutic approaches [20, 21, 22, 23, 24, 25, 26, 27, 28, 29]. In a clinical setting, real‐time tracking of TAMs phenotype in vivo would be critical for obtaining real‑time feedback on treatment response and enabling timely adjustments of therapeutic regimens [30, 31].

Currently, the assessment of TAMs phenotype predominantly relies on ex vivo or end‐point assays, such as flow cytometry, histological (immunohistochemistry or immunofluorescence), and bulk or single‐cell RNA sequencing, to quantify surface markers, transcriptional programs, or functional status [32, 33]. While these methods provide in‐depth molecular and phenotypic detail, they require invasive tissue sampling or cell isolation and are incapable of capturing the spatiotemporal dynamics of TAM phenotypic changes in living subjects. This limitation restricts their utility for pharmacodynamics, adaptive dosing strategies and longitudinal monitoring during drug development or clinical treatment. Noninvasive imaging modalities (PET/SPECT, MRI, etc.) have been developed to monitor TAMs in vivo using targeted probes [30, 34, 35]. However, a broadly applicable, high‐sensitivity method capable of in vivo quantification of TAM phenotypic transition has not yet been achieved. Therefore, developing visualization methods that can dynamically monitor TAM phenotypes in vivo is crucial for accelerating the rational design and clinical translation of TAM‑targeted therapies. Near‐infrared‐II (NIR‐II) fluorescence imaging technology has the advantages of high temporal and spatial resolution and high tissue penetration depth, and can visualize biological processes in real‐time and non‐invasively [36, 37]. NIR‐II small molecule photoacoustic (PA) imaging combines the advantages of photoexcitation and acoustic detection, and can provide three‐dimensional (3D) images, which makes it possible to sensitively detect low‐abundance biomarkers during TAMs reprogramming [38].

**SCHEME 1:**
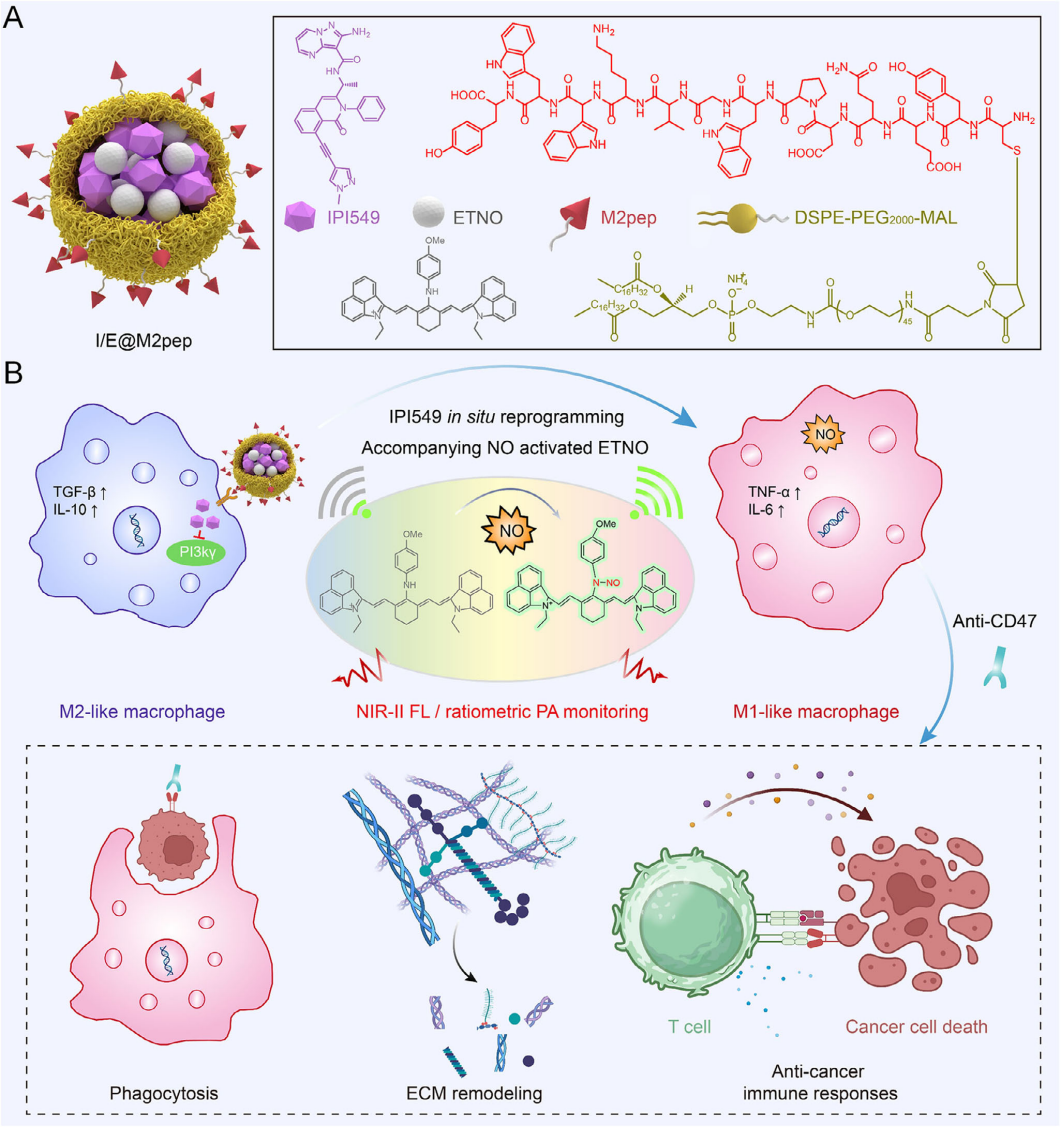
Schematic illustration of I/E@M2pep components (A) and in vivo visualizing TAMs reprogramming for tumor treatment (B).

Herein, we designed an NO‐activatable NIR‐II nanoinducer (I/E@M2pep) for real‐time in vivo visualization of TAM reprogramming while simultaneously enhancing anti‑tumor efficacy (Scheme 1). I/E@M2pep comprises three functional units: 1) a M2 macrophage‑targeting peptide (M2pep)‐conjugated DSPE‐PEG amphiphilic moiety that facilitates nanoparticle self‐assembly and receptor‑mediated uptake by M2 TAMs [39, 40]; 2) a small‑molecule drug IPI549 that reprograms M2 TAMs toward the M1 phenotype by inhibiting PI3Kγ signaling [25, 41]; and 3) an NO‑activatable NIR‐II fluorophore (ETNO) that enables in vivo visualization of M2‐to‐M1 TAM repolarization by ratiometric PA imaging. After systemic administration, I/E@M2pep preferentially accumulates in tumors through receptor‑mediated uptake by M2 TAMs. Following release, IPI549 reprograms M2 TAMs into an M1‐like phenotype, and the NO production from M1 TAMs modulates the ratiometric PA signal of ETNO, thereby enabling real‐time imaging of macrophage reprogramming in vivo. In a mouse breast cancer model, the nanoinducer effectively repolarized TAMs into an M1‐like phenotype and significantly improved anti‐tumor efficacy, particularly when combined with a CD47 blockade monoclonal antibody (CD47 mAb). This study establishes a theranostic platform for effective TAMs repolarization and its real‐time monitoring in vivo, which can be extended to investigate the role of macrophages in tumor initiation, metastasis, and therapeutic response, thereby providing a framework for the precise diagnosis and treatment of tumors.

## 2 Results and Discussion

### 2.1 Design and Characterization of I/E@M2pep

**FIGURE 1:**
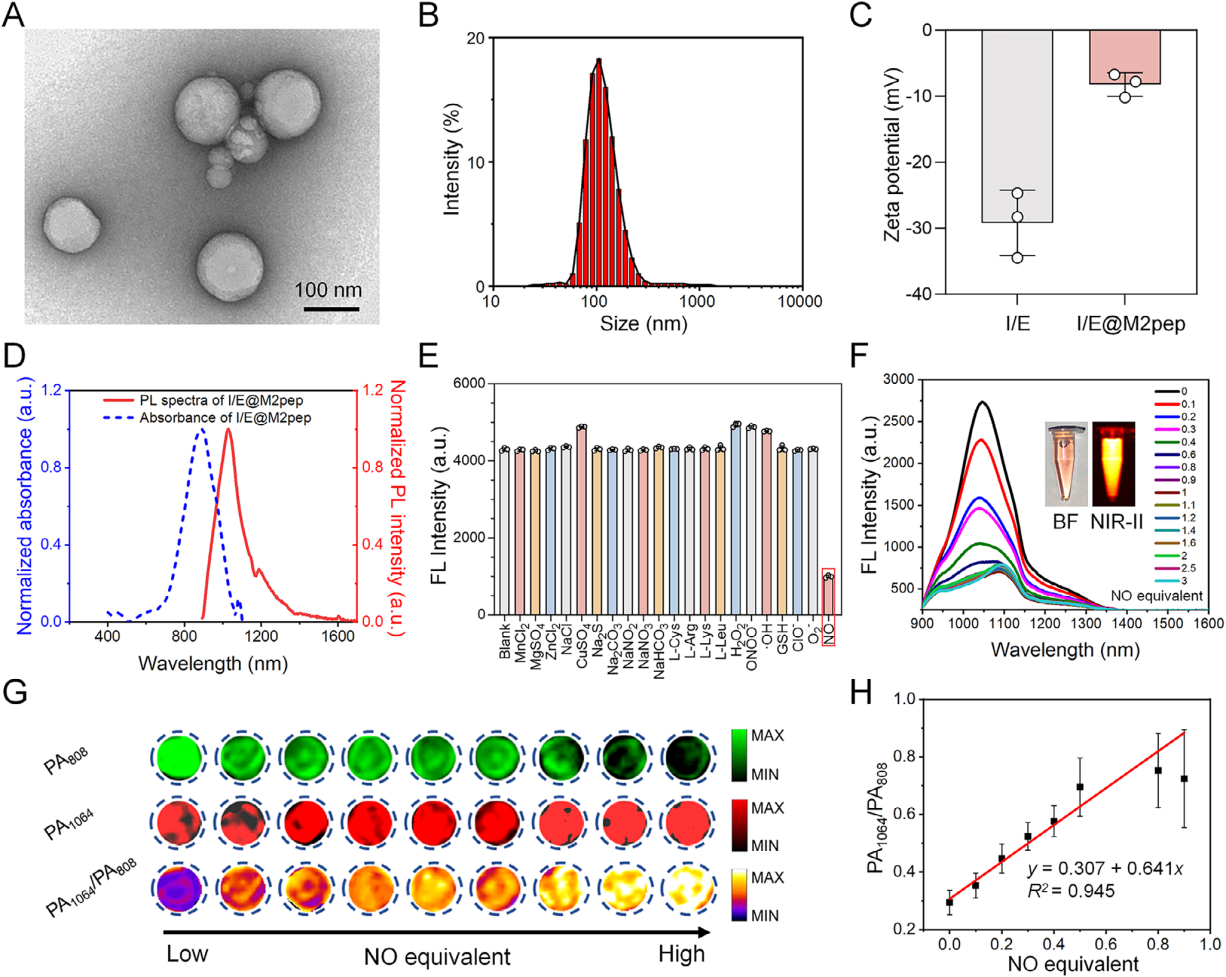
Characterization of I/E@M2pep. (A) TEM image of I/E@M2pep. Scale bar: 100 nm. (B) Dynamic light scattering of I/E@M2pep. (C) Zeta potential of I/E and I/E@M2pep (*n*=3). The error bars indicate SD. (D) The absorption and emission of I/E@M2pep. (E) The NO fluorescence selectivity of I/E@M2pep (*n*=3). The error bars indicate SD. (F) The fluorescence spectra of I/E@M2pep incubation with different concentrations NO. (G) PA images of I/E@M2pep incubation with different concentrations NO. (H) The ratiometric PA signal of the I/E@M2pep incubation with different concentrations NO (*n*=3). The error bars indicate SD.

Inducible NO synthase (iNOS) is a hallmark molecule of M1 macrophages, which can synthesize NO by using arginine as substrate [42, 43]. We therefore selected NO as an ideal target for identifying M1 macrophages and synthesized the NO‐activatable NIR‐II probe ETNO. The chemical structure of ETNO was confirmed by ^1^H NMR, ^13^C NMR, and mass spectra (Figures S1–S3). ETNO shows a maximum absorption at 887 nm and emission at 1045 nm in DMSO (Figure S4). The molar extinction coefficient of ETNO is 2.627 × 10^5^ M^−1^ cm^−1^, and the quantum yield is 0.023% (Figures S5 and S6). After the specific reaction between ETNO and NO, the intramolecular charge transfer (ICT) effect was blocked and the optical properties changed (Figures S7–S9). I/E@M2pep was obtained by self‐assembly of IPI549, ETNO, and DSPE‐PEG_2000_‐MAL, followed by functionalization with the targeting peptide M2pep. The encapsulation efficiency of ETNO and IPI549 was determined to be 62% and 58%, respectively (Figure S10). I/E@M2pep presents a regular spherical structure with a particle size of about 110 nm (Figure 1A,B). Zeta potential results show that I/E exhibits a surface potential of −34.5 mV, which is related to maleimide groups on the surface of DSPE‐PEG micelles. After linking M2pep to the micelle surface, its surface potential increased to −7.8 mV (Figure 1C). The initial absorption peak of I/E@M2pep is at 883 nm and the emission peak is at 1022 nm (Figure 1D). Due to the outer PEG layer, the nanoinducers exhibited excellent stability in various solvents (H_2_O, PBS, FBS, or DMEM) and across a wide pH range from pH 4 to 9 (Figures S11 and S12).

The specific response to NO is fundamental for assessing and monitoring of TAMs reprogramming. We therefore tested a variety of ions and amino acid solutions to evaluate the selective response of I/E@M2pep to NO. The fluorescence intensity of I/E@M2pep decreased only after treatment with NO, while no apparent changes were observed in the other groups (Figure 1E). Meanwhile, the emission spectra of I/E@M2pep gradually decreased with the increase of NO concentration (Figure 1F; Figure S13). As NO content increased, the absorption peak of I/E@M2pep at 808 nm gradually decreased while a new absorption peak appeared at 1064 nm (Figure S14). This spectral change enables quantification of NO by absorption ratio Abs_1064 nm_/Abs_808 nm_, with a detection limit down to 0.19 µM (Figure S14). After five cycles of photothermal heating and cooling, I/E@M2pep retained good photothermal stability, guaranteeing its use for PA imaging (Figure S15). We further measured the PA signals of I/E@M2pep under 808 nm and 1064 nm irradiation and found that the ratiometric PA signal (PA_1064 nm_/PA_808 nm_) correlated linearly with NO concentration (Figure 1G,H; Figure S16). Thus, changes in the ratiometric PA signal can be used to monitor NO generation and dynamically evaluate the therapeutic response during TAM reprogramming.

### 2.2 Reprogramming and Monitoring Macrophage Using I/E@M2pep In Vitro

**FIGURE 2:**
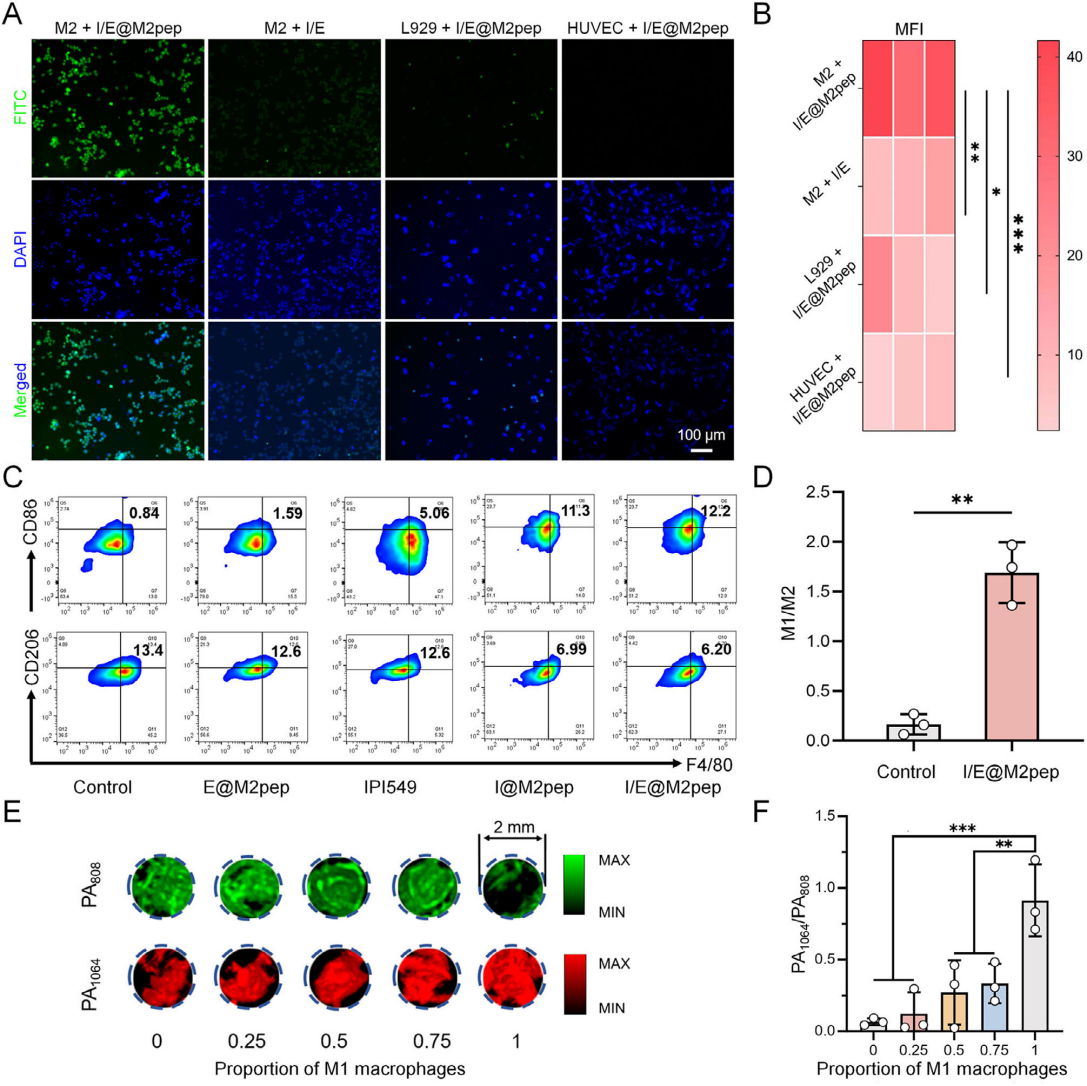
Reprogramming and detection capabilities of I/E@M2pep in vitro. (A) Fluorescence images of cellular uptake of I/E@M2pep in different cell lines. Scale bar: 100 µm. (B) Fluorescence intensity analysis of different groups in A (*n*=3). (C) Dynamic changes of two macrophage subsets after different treatment in BMDMs by flow cytometry analysis. (D) Quantification of M1/M2 rate following the treatment of I/E@M2pep in BMDMs (*n*=3). (E) PA images of different contents of M1 macrophages incubated with I/E@M2pep. (F) Correlation studies between percentages of M1 macrophages and PA_1064 nm_/PA_808 nm_ (*n*=3). Statistical significance: All data are presented as the mean ± SD. *p < 0.05, **p < 0.01, ***p < 0.001, as determined by unpaired *t*‐test (D) or one‐way ANOVA (B and F).

In order to minimize the interference of NO produced by non‐target cells (endothelial cells and neutrophils) in the TME, we functionalized the outermost layer of the nanoinducer with M2pep to target M2 macrophages. We first assessed the targeting ability of I/E@M2pep to TAMs in vitro. FITC‐labeled nanoinducer, with or without the M2pep, was co‐incubated with different cells for 30 min. As shown in Figure 2A,B, the M2pep modification enabled I/E@M2pep to specifically target and accumulate in M2 macrophages, whereas the unmodified nanoinducer showed no obvious targeting. This specificity was further confirmed by minimal uptake in other cell types (Figures S17 and S18). To verify the reprogramming effect of the nanoinducer, RAW264.7 cells and murine bone marrow‐derived macrophages (BMDMs) were pre‐polarized to the M2 phenotype, then incubated with nanoinducers. The macrophage phenotype was analyzed by flow cytometry after 24 h. Compared with other treatment groups, cells treated with I/E@M2pep showed a significant increase in M1 markers and a corresponding decrease in M2 markers (Figure 2C; Figures S19–S21). The M1/M2 ratio of macrophages in the I/E@M2pep‐treated group increased by 10.4 times (Figure 2D). The above results confirmed the efficient targeting and reprogramming effect of I/E@M2pep on M2 macrophages in vitro.

To determine the relationship between the nanoinducer's ratiometric PA response and the M1/M2 composition, I/E@M2pep was incubated with macrophage mixtures of varying M1:M2 ratios for 8 h, followed by ratiometric PA imaging. As expected, in a pure M2 environment the probe was not triggered because of the negligible NO level, producing a very low PA_1064 nm_/PA_808 nm_ ratio (≈ 0.08). In contrast, in a pure M1 environment the probe was clearly activated by NO, with the ratiometric signal approaching 1 (Figure 2E,F). These results indicate that changes in the ratiometric PA signal can be used to monitor macrophage repolarization in real‐time.

### 2.3 In Vivo FL/PA Dual‐Modal Imaging of M2 Macrophage Reprogramming

**FIGURE 3:**
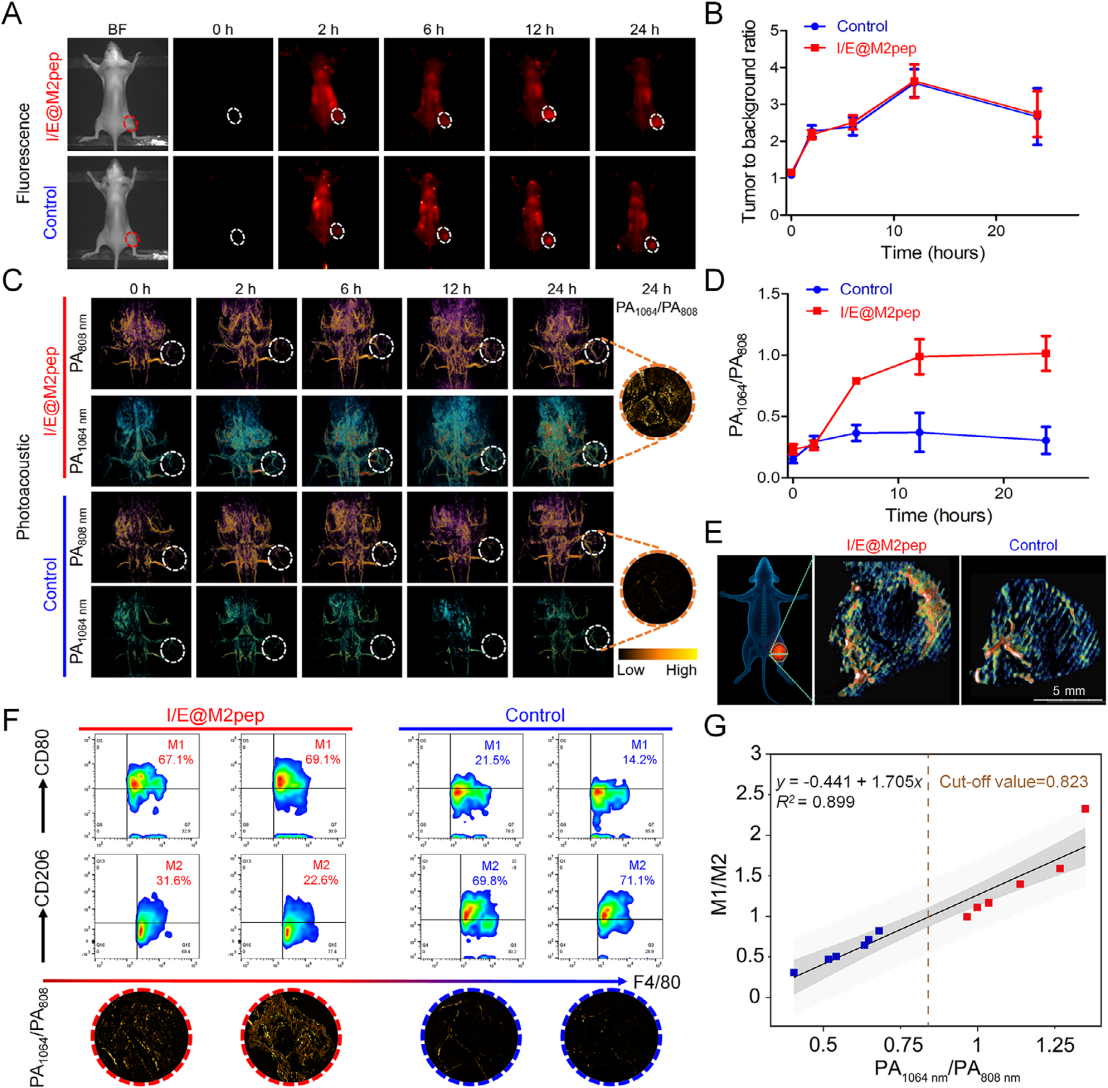
In vivo real‐time FL/PA imaging of TAMs. (A) Real‐time NIR‐II fluorescence imaging in 4T1‐bearing mice after intravenous injection of I/E@M2pep or E@M2pep (control group). (B) Quantification of tumor NIR‐II fluorescence signal in different groups (*n*=3). The error bars indicate SD. (C) Real‐time PA imaging in 4T1‐bearing mice after intravenous injection of I/E@M2pep. (D) Quantification of ratiometric PA signal from the 4T1‐bearing mice in different groups (*n*=3). The error bars indicate SD. (E) 3D PA imaging of the largest cross‐sections of the tumors treated with the nanoinducers after 24 h. Scale bar: 5 mm. (F) Flow cytometry analysis of M1 and M2 macrophages proportion in tumor tissues from each group and the corresponding ratiometric PA tumor image. (G) Correlation between M1/M2 and PA_1064 nm_/PA_808 nm_ in both groups. The cut‐off value of 0.823 was calculated as the optimal threshold through receiver operating characteristic (ROC) curve analysis.

Since the PA signal of I/E@M2pep sensitively respond to NO in the reprogramming process of TAMs in vitro, we further evaluated its feasibility in vivo. In a 4T1 tumor‐bearing mouse model, upon intravenous administration, I/E@M2pep gradually accumulates in tumors (Figure 3A; Figure S22). Quantification of FL signal intensity ratios showed that tumor to background ration increased over time after nanoinducer injection, reaching a maximum at 6 h (Figure 3B). Under 808 nm excitation, PA signal intensities at tumor sites were similar regardless of whether mice received the reprogramming drug. However, with 1064 nm excitation, tumors from drug‐treated mice exhibited substantially stronger PA signals than untreated controls, indicating that NO produced by reprogrammed M1 macrophages reacted with ETNO and thereby activated the 1064 nm PA signal (Figure 3C,D). Furthermore, through three‐dimensional (3D) PA imaging, we demonstrated the capability of the nanoinducer to monitor M1‐like macrophage infiltration across different tumor depth cross‐sections in real time (Figure 3E).

To assess the relationship between ratiometric PA signals (PA_1064 nm_/PA_808 nm_) from activated ETNO and macrophage repolarization, we performed flow cytometry on tumor tissues. In 4T1 tumor‐bearing mice receiving IPI549 reprogramming treatment, tumor infiltration by M1 macrophages increased approximately 3‐fold compared with non‐reprogrammed controls, while M2 TAMs decreased roughly 2‐fold, consistent with IPI549‐induced conversion of M2 to M1 (Figure 3F). Concurrently, the ratiometric PA signal (PA_1064 nm_/PA_808 nm_) at tumor sites correlated linearly with the M1/M2 ratio (Figure 3G). Moreover, the cut‐off values were determined to be 0.823, which can accurately evaluate the repolarization effect of macrophages through the ratiometric PA signal (Figure 3G; Figure S23). These findings confirm the nanoinducer's potential for in vivo real‐time tracking of TAM reprogramming.

### 2.4 I/E@M2pep Reconstruct TME to Potentiate Anti‐Tumor Effect

**FIGURE 4:**
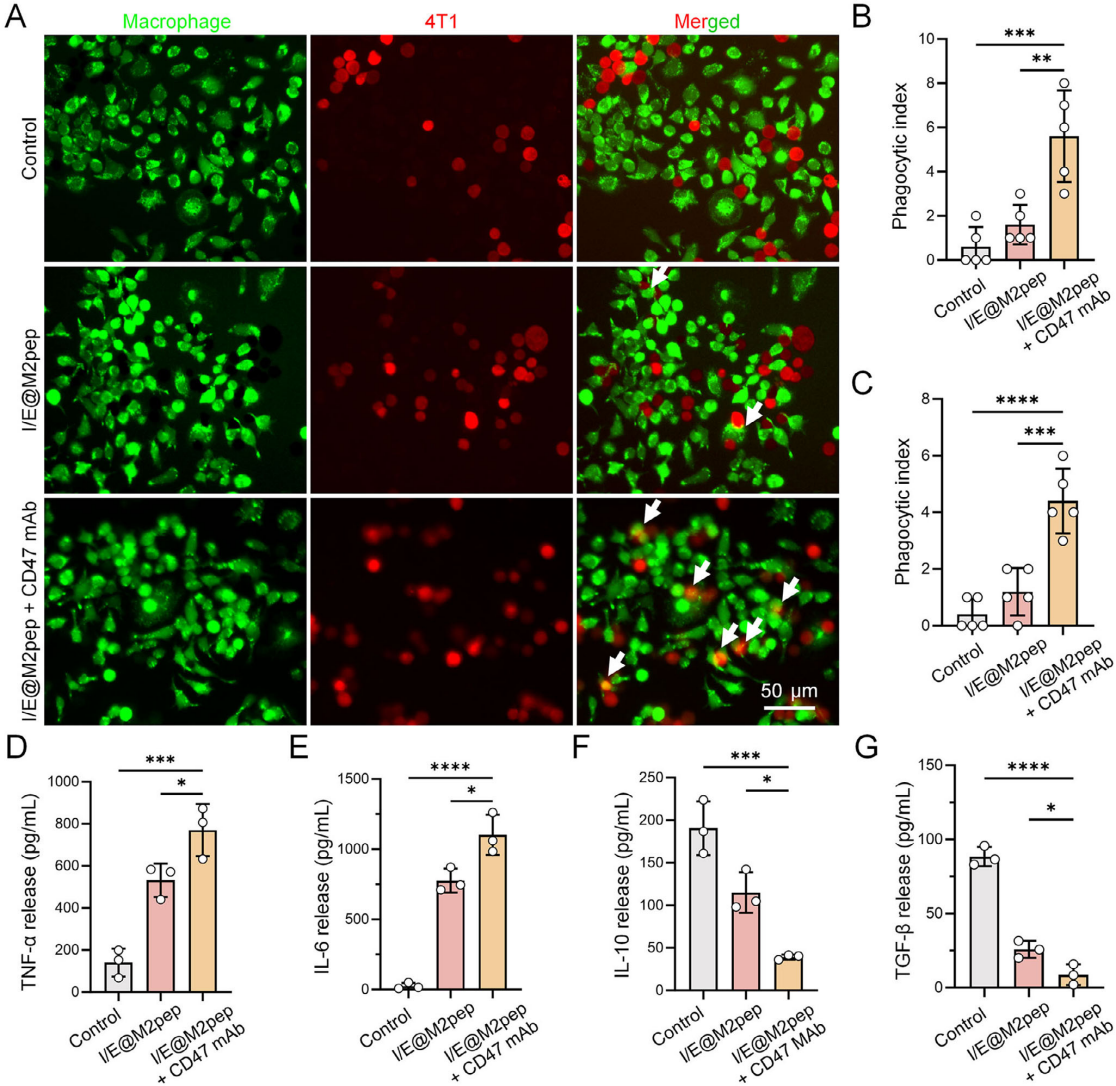
Evaluation of the phagocytosis of tumor cells by reprogrammed macrophages. (A) Representative images of macrophages (RAW264.7) phagocytosing tumor cells following treatment with I/E@M2pep with or without CD47 mAb. Arrows point to phagocytosed tumor cells. Scale bar: 50 µm. (B) Phagocytic index of RAW264.7 in different groups (*n*=5). (C) Phagocytic index of BMDM in different groups (*n*=5). (D–G) The concentrations of TNF‐α (D), IL‐6 (E), IL‐10 (F) and TGF‐β (G) in the supernatant of macrophages co‐cultured with 4T1 cells treated with I/E@M2pep combined with CD47 mAb at an E/T ratio of 1:1 (*n*=3). Statistical significance: All data are presented as the mean ± SD. *p < 0.05, **p < 0.01, ***p < 0.001, ****p < 0.0001, as determined by one‐way ANOVA (B‐G).

The TAM phenotype shifted from M2 to M1, resulting in a functional switch from promoting tumor progression to inhibiting it. First, we evaluated the phagocytosis of reprogrammed macrophages on tumor cells and the changes in cytokine secretion level in vitro. Because tumor cells highly express the CD47 “do not eat me” signal, we included an experimental group receiving CD47 mAb. The results showed that phagocytosis by macrophages reprogrammed with I/E@M2pep was significantly enhanced when combined with CD47 mAb (Figure 4A–C; Figure S24). ELISA quantification of M1‐associated cytokines, including TNF‐α and IL‐6, revealed significant increases following I/E@M2pep treatment (Figure 4D–G; Figure S25). Meanwhile, levels of immunosuppressive molecules IL‐10 and TGF‐β decreased, which should help relieve the ECM barrier and improve anti‐tumor immune responses.

**FIGURE 5:**
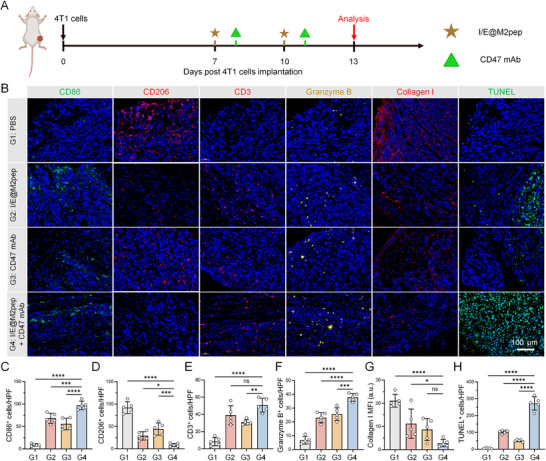
Remolding the TME by I/E@M2pep combined with CD47 mAbs to potentiate anti‐tumor effect. (A) Schematic illustration of the treatment protocol. (B) CD86, CD206, CD3, Granzyme B, collagen I, and TUNEL staining of tumor tissue in each treatment group. Scale bar: 100 µm. (C) Statistical analysis was conducted on the count of CD86‐positive cells under each high‐power field (HPF, 200 ×) (*n*=5). (D) Statistical analysis was conducted on the number of CD206‐positive cells under each HPF (*n*=5). (E) Statistical analysis was conducted on the number of CD3‐positive cells under each HPF (*n*=5). (F) Statistical analysis was conducted on the number of granzyme B‐positive cells under each HPF (*n*=5). (G) Mean fluorescence intensity (MFI) analysis of collagen type I under each HPF (*n*=5). (H) Statistical analysis was conducted on the number of TUNEL‐positive cells under each HPF (*n*=5). Statistical significance: All data are presented as the mean ± SD. *p < 0.05, **p < 0.01, ***p < 0.001, ****p < 0.0001; ns: not significant, as determined by one‐way ANOVA (C–H).

Based on the excellent macrophage‐reprogramming efficacy of I/E@M2pep, we further investigated its effects on TME remodeling and anti‐tumor activity in vivo. The results showed that the M2 macrophage marker CD206 decreased significantly and the M1 macrophage marker CD80 increased significantly in I/E@M2pep treatment group (Figure 5A–D). When combined with CD47 mAb, the effect is more significant. The decrease of collagen expression in the combined treatment group was accompanied by the infiltration of T cells and the increase of Granzyme B, which indicated that the anti‐tumor immune response was significantly improved (Figure 5B,E–G). Histological analyses further confirmed increased apoptosis in the nanoinducer‐treated combined with CD47 mAb groups (Figure 5B,H). Collectively, these results indicate that I/E@M2pep combined with CD47 mAb remodels the TME via in situ macrophage reprogramming, thereby potentiating anti‐tumor immunity.

**FIGURE 6:**
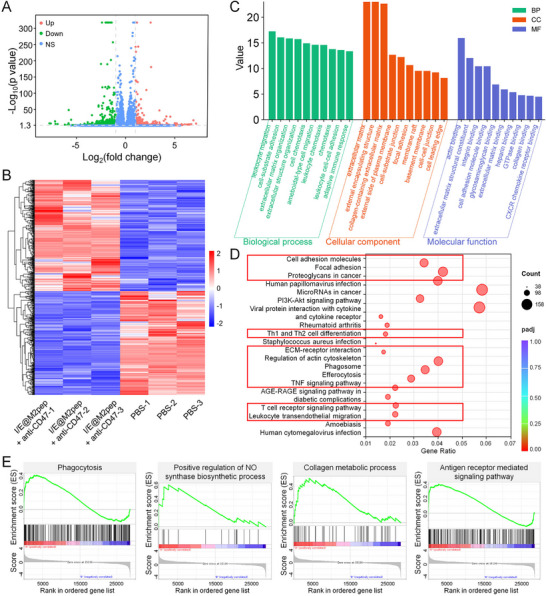
RNA‐seq analysis of the potential mechanism of remolding the TME by I/E@M2pep combined with CD47 mAbs. (A) Volcano plot of differential gene expression profiles induced by I/E@M2pep combined with CD47 mAbs. (B) Heat map of RNAseq transcriptome analysis for differentially expressed genes. (C) Top 10 enriched GO terms related to anti‐tumor immune response, cell migration, and extracellular matrix remodeling. (D) Top 20 pathways identified through KEGG enrichment assay. In the red boxes are pathways related to anti‐tumor immune response and extracellular matrix remodeling. (E) GSEA enrichment plots showing that pathways of phagocytosis, NO synthesis, collagen metabolism, and anti‐tumor immune response are upregulated in the I/E@M2pep combined with CD47 mAbs group.

We further evaluated TME changes induced by the combination therapy using RNA sequencing (RNA‐seq) of tumor tissues. Compared with the PBS group, the I/E@M2pep + CD47 mAb group showed 474 upregulated and 444 downregulated genes (Figure 6A,B). Gene Ontology (GO) analysis revealed significant enrichment of terms related to cell adhesion, leukocyte migration, ECM organization, and related processes (Figure 6C). KEGG pathway analysis of the top 20 pathways highlighted immune cell migration, anti‐tumor immune responses, and ECM remodeling, further supporting that TME remodeling improves anti‐tumor immunity (Figure 6D). Importantly, the gene set enrichment analysis (GSEA) demonstrated notable enrichment of phagocytosis, NO synthesis, collagen metabolism, and anti‐tumor immune response, all of which were upregulated in the combined group (Figure 6E). Overall, RNA seq results indicate that I/E@M2pep combined with CD47 mAb may enhance tumor eradication by remodeling the TME and potentiating anti‐tumor immune responses. Additionally, no obvious histopathological toxicity was observed in major organs (heart, liver, spleen, lung, and kidneys), and hematologic toxicity was negligible, supporting the good biosafety of the nanoinducer (Figures S26–S28).

## 3 Conclusions

In summary, we developed a novel NO‐activatable NIR‐II nanoinducer (I/E@M2pep) for in vivo visualizing reprogramming of TAMs and enhancement of solid tumor therapy. I/E@M2pep was prepared by facile self‐assembly. M2pep confers selective targeting of M2 TAMs; the PI3Kγ inhibitor IPI549 induces conversion of M2 TAMs to an M1‐like phenotype accompanied by NO production; and the ETNO probe enables in vivo visualization of macrophage repolarization via NIR‐II fluorescence/photoacoustic imaging. The target‐activated, deep‐tissue‐penetrating NIR‐II imaging strategy markedly enhanced the specificity and sensitivity of in vivo detection, yielding a high signal‐to‐background ratio. Through the ratiometric PA signal, we can accurately evaluate the repolarization effect of macrophages. Moreover, combining the nanoinducer with a CD47 mAb boosted anti‐tumor immunity via M1 macrophage‐mediated tumor killing and TME remodeling. This strategy can be extended to monitor tumor initiation, metastasis, and therapeutic response, thereby helping to clarify the roles of TAMs in tumor progression and treatment outcomes.

## 4 Experimental Section

### 4.1 Preparation and Characterization of I/E@M2pep

The preparation and characterization of the probe I/E@M2pep were conducted as follows. The probe was synthesized using a classical self‐assembly and solvent evaporation method. Specifically, IPI549 (0.5 mg), ETNO (1.2 mg), DSPE‐PEG_2000_ (8 mg), and DSPE‐PEG_2000_‐MAL (2 mg) were dissolved in 5 mL of THF and ultrasonicated for 3 min to achieve homogeneous dispersion. This mixture was then slowly injected into 20 mL of vigorously stirred deionized water at room temperature, followed by continuous stirring for 1 h. The resulting solution was concentrated using a 30 kDa ultrafiltration tube to remove residual THF. To confer M2 macrophage targeting capability, M2pep peptide (sequence: YEQDPWGVKWWYC) was conjugated to the micelle surface. The peptide was dissolved in 2 mL DMSO and gradually introduced into the 10 mL micelle solution under constant stirring for 4 h. Post‐conjugation, the solution underwent additional ultrafiltration (30 kDa) and dual filtration through 0.22 µm membranes to eliminate unencapsulated components. Physicochemical characterization included dynamic light scattering (Malvern Zetasizer Nano ZS) for hydrodynamic diameter and zeta potential measurements, transmission electron microscopy (HT7700) for morphological analysis, and UV–vis‐NIR/fluorescence spectroscopy for optical property assessment.

Encapsulation efficiency (EE) was determined through a three‐step protocol: 1) Establishing standard curves for IPI549 and ETNO using methanol solutions of graded concentrations; 2) Disrupting micelles via 10 min high‐intensity ultrasonication of 20 µL probe solution in 980 µL methanol; 3) Calculating actual drug content from absorbance measurements at 360 nm (IPI549) and 890 nm (ETNO) against standard curves. This revealed EE values of 58% for IPI549 and 62% for ETNO.

### 4.2 The Selective Reactivity of I/E@M2pep Toward Different Analytes

The fluorescence response selectivity of I/E@M2pep toward different analytes was evaluated by testing its interactions with various ions, amino acids, and reactive oxygen species. Specifically, solutions of H_2_O_2_ (0.4–400 µM), ONOO^−^ (50 µM), ·OH (50 µM), Na^+^, Mn^2+^, Zn^2+^, Cu^2+^, Mg^2+^, HCO_3_^−^, CO_3_^2−^, NO_3_^−^, NO_2_^−^, S^2−^, L‐Cys, L‐Arg, L‐Lys, L‐Leu, GSH (all 50 µM), and NO (0.4–400 µM) were prepared using standardized protocols. Probe solutions (10 µM) were mixed with each analyte solution at a 1:1 volume ratio (final molar ratio 1:5) and incubated at 37°C for 10 min under ultrasonic homogenization. Fluorescence intensity at 1045 nm was measured using a near‐infrared spectrometer (808 nm excitation), while photoacoustic signals were acquired at 808 and 1064 nm wavelengths using a LOIS VIEW 3D imaging system (LOIS‐3D, TomWave) with dedicated solution‐imaging molds.

### 4.3 Evaluation of no Response of I/E@M2pep In Vitro

The NO responsiveness of the probe was quantitatively evaluated through gradient concentration tests. DEA NONOate (NO donor) solutions were freshly prepared in ice‐cold PBS with concentrations ranging from 0.4 to 400 µM (14 gradients). The probe solution (40 µM in acetonitrile) was added to pre‐warmed NO solutions (37°C) at 1:1 volume ratio. After 1 min equilibration, absorption spectra (400–1100 nm) and fluorescence spectra (900–1500 nm) were recorded using UV–vis‐NIR spectrophotometer (Shimadzu UV‐3600) and NIR fluorescence spectrometer (Edinburgh FLS1000) respectively. For photoacoustic characterization, 200 µL of probe‐NO mixtures were injected into optically transparent latex tubes and imaged using LOIS 3D PA imaging system (VevoLazer 3100) with laser wavelengths spanning 700–1064 nm. The PA_1064 nm_/PA_808 nm_ signal was calculated to establish quantitative correlation. Detection limit was determined via linear regression of absorbance ratio (Abs_1064 nm_/Abs_808 nm_) versus NO concentration (0–0.5 eq).

### 4.4 Cell Culture and Animal Tumor Model

All animal experiments were conducted in compliance with relevant laws and approved by the Animal Care and Use Committee of SINANO (assigned approval number: SINANO/EC/2023–021). Female BALB/c mice, aged 5 weeks and weighing 18‐20 g, were purchased from GemPharmatech. 4T1 cells (RRID: CVCL_0125), RAW 264.7 cells (RRID: CVCL_0493), L929 cells (CVCL_0462) and HUVEC cells (CVCL_2959) were purchased from the Cell Bank of the Chinese Academy of Sciences. These cells were cultured in DMEM or RPMI 1640 supplemented with 10% FBS and incubated at 37°C with 5% CO_2_. A tumor model in female BALB/c mice was established by subcutaneous implantation of 1 × 10^6^ 4T1 cells in 100 µl PBS. Tumor treatment and imaging experiments were initiated when the tumor volume reached 60–80 mm^3^. The maximum tumor volume did not exceed the ethical requirements.

### 4.5 Flow Cytometry Analysis

For flow cytometric analysis of macrophage polarization states, single‐cell suspensions derived from bone marrow were stained with fluorochrome‐conjugated antibodies under physiological conditions (37°C, 5% CO_2_). The staining protocol included anti‐F4/80 for macrophage gating, coupled with CD80‐APC and CD206‐PE‐Cy5 to differentiate M1 and M2 subtypes. After 30 min incubation, cells underwent three PBS washing cycles to eliminate unbound antibodies prior to analysis on a Beckman Cytoflex flow cytometer. For *ex vivo* assays, M1 (CD45^+^, CD11b^+^, F4/80^+^, CD80^+^ or CD86^+^) and M2 (CD45^+^, CD11b^+^, F4/80^+^, CD206^+^) macrophages were analyzed by FACS from single‐cell suspensions prepared from subcutaneous xenograft tumors established in Balb/C mice.

### 4.6 In Vitro Macrophage Reprogramming Assays

To establish the correlation between the ratio‐based photoacoustic signals and M1/M2 macrophage proportions, in vitro macrophage assays were conducted using bulk cell populations. Specifically, 10^8^ M0‐type macrophages were harvested and polarized into M1 and M2 subtypes (5 × 10^7^ cells each). These polarized populations were then mixed at defined M1 ratios (0%, 25%, 50%, 75%, and 100%), with each mixture containing approximately 2 × 10^7^ macrophages. The cell mixtures were incubated with 15 µM probe solution for 1 h, followed by 8 h culture in serum‐free medium to allow cellular response. Post‐incubation procedures included cell dissociation through enzymatic digestion, PBS washing (twice), and resuspension in 300 µL of pre‐warmed 1% agarose solution. The cell‐agarose mixtures were rapidly injected into latex tubes for physical immobilization, forming macroscopic cellular aggregates visible to the naked eye. Photoacoustic signal acquisition was performed using standard solution imaging protocols, with subsequent data analysis focused on generating bar chart comparisons of photoacoustic signals across different M2 ratio groups.

### 4.7 In Vivo Phagocytosis Function Analysis

For in vitro phagocytosis function analysis, macrophages were plated in confocal cell culture dishes. After the cells adhered to the wall, IL‐4 and IL‐13 were used to induce them to differentiate into M2 macrophages, and then I/E@M2pep was added for reprogramming treatment. According to the manufacturer's protocol, the macrophages were labeled with Calcein before co‐incubation with 4T1 cells expressing the fluorescent protein mCherry. Then add the same number of living tumor cells. Add or not add CD47 mAb (10 µg mL^−1^) and incubate at 37°C for 4 h. The cells were washed repeatedly, and then imaged by confocal microscope. The phagocytosis index was calculated as the number of mCherry^+^ cells swallowed per 100 macrophages.

### 4.8 Analysis of Cytokine Production

Reprogrammed macrophages and 4T1 cells were co‐cultured with or without CD47 mAb for 24 h. The co‐culture supernatant was collected and the secretion level of cytokines was analyzed by ELISA. The concentrations of TNF‐α, IL‐6, IL‐10, and TGF‐β were measured using commercially available ELISA kits (Real‐gen Biotechnology) according to the manufacturer's recommended protocols.

### 4.9 In Vivo Real‐Time NIR‐II Fluorescence/Photoacoustic Imaging

For fluorescence imaging, mice were anesthetized with 2% isoflurane and placed in a prone position on a heating pad maintained at 37°C. I/E@M2pep (40 µM in PBS, 200 µL) was intravenously injected via the tail vein. Real‐time NIR‐II fluorescence signals (900–1500 nm) were acquired using a custom‐built imaging system equipped with an 808 nm laser (1 W/cm^2^) and an InGaAs camera (Princeton Instruments), with exposure time set to 100 ms and binning factor of 4 × 4. Fluorescence images were captured at 0, 2, 6, 12, and 24 h post‐injection, followed by spectral unmixing to eliminate tissue autofluorescence. For ratiometric photoacoustic imaging, an integrated photoacoustic‐ultrasound system (LOIS‐3D, TomWave) was employed with dual‐wavelength excitation (808 nm and 1064 nm). The laser energy density was calibrated to 10 mJ/cm^2^ using a power meter (Ophir Nova II). Photoacoustic signals were acquired through a 128‐element ultrasound transducer array (central frequency: 5 MHz, bandwidth: 60%) and reconstructed using delay‐and‐sum algorithms. Ratiometric analysis (PA_1064 nm_/PA_808 nm_) was performed to quantify nitric oxide levels, with region‐of‐interest (ROI) selection guided by co‐registered ultrasound images. All imaging data underwent three‐dimensional reconstruction and signal normalization against baseline measurements.

### 4.10 Immunofluorescent Staining

Tumors from each group were harvested and embedded in cryo‐embedding media (OTC compound) to generate cryo‐sections with a thickness of 10 µm. The tissue sections were then fixed in a fixative solution for 30 min, followed by washing with PBS. Subsequently, the sections were blocked with 5% bovine serum albumin for 30 min at room temperature. After another PBS wash, the sections were incubated with a primary antibody overnight at 37°C. The sections were then incubated with a secondary antibody for 60 min at 37°C. Nuclei were stained with DAPI for 15 min. Fluorescence images were captured using a confocal laser scanning microscope.

### 4.11 Rna Sequencing (RNAseq)

The fresh tumor tissues were collected after two doses for RNAseq. Under a transfer agreement, the samples were transferred to Novogene (Beijing, China) for RNA‐seq, which was performed on an Illumina Novaseq platform.

### 4.12 Statistical Analysis

Data were analyzed using GraphPad Prism 10.1 software. Data are expressed as mean ± standard deviation (SD). Statistical significance was determined using student's t test or one‐way analysis of variance (ANOVA), one‐way ANOVA with Tukey's post‐hoc test were employed for multiple group comparisons where applicable, with *p < 0.05, **p < 0.01, ***p < 0.001 and ****p < 0.0001 were considered statistically significant.

## Conflicts of Interest

The authors declare no conflicts of interest.

## Supporting information




**Supporting File**: advs74774‐sup‐0001‐SuppMat.docx.

## Data Availability

The data that support the findings of this study are available in the supplementary material of this article.
