# Supplementary material for: Real‐Time In Vivo Visualization of Tumor‐Associated Macrophage Reprogramming Using a Nitric Oxide‐Activatable NIR‐II Nanoinducer
Source: Adv Sci (Weinh). 2026 Mar 10;13(29):e24367. doi: 10.1002/advs.202524367 (PMC13205585; doi:10.1002/advs.202524367)
Supplement: Supplementary file 1 — Supporting File: advs74774‐sup‐0001‐SuppMat.docx. [file ADVS-13-e24367-s001.docx]

**Supporting Information**

Real-Time *In Vivo Visualization of* Tumor-Associated Macrophage Reprogramming Using a Nitric Oxide-Activatable NIR-II Nanoinducer

Qian Chen^+[a,b]^, Meng Li^+[a]^, Tuanwei Li^+[a]^, Chen Yang^[a]^, Xiaohu Yang^[a]^, Hongchao Yang^[a]^, Yejun Zhang^[a]^, Chunyan Li*^[a]^, and Qiangbin Wang^[a]^

[a] Dr. Q. Chen, M. Li, Dr. T. Li, C. Yang, Dr. X. Yang, Dr. H. Yang, Dr. Y. Zhang, Prof. C. Li, Prof. Q. Wang
CAS Key Laboratory of Nano-Bio Interface
Suzhou Key Laboratory of Functional Molecular Imaging Technology
Division of Nanobiomedicine and *i*-Lab
Suzhou Institute of Nano-Tech and Nano-Bionics
Chinese Academy of Sciences
Suzhou 215123, China

[b] Dr. Q. Chen
Medical Science and Technology Innovation Center

The Affiliated Suzhou Hospital of Nanjing Medical University

Suzhou Municipal Hospital

Gusu School of Nanjing Medical University
 Suzhou 215000, China

[+] These authors contributed equally to this work.

*E-mail: cyli2012@sinano.ac.cn (C. Li)

**1. Experimental Materials**

**Table S1. Key reagent or resource**

| Number | Reagent or resource | Source |
| --- | --- | --- |
| 1 | RPMI 1640 | HyClone |
| 2 | DMEM | HyClone |
| 3 | FBS | Gibco |
| 4 | Penicillin-Streptomycin Solution | Beyotime |
| 5 | Trypsin-EDTA Solution | Beyotime |
| 6 | Hoechst 33342 | Beyotime |
| 7 | Calcein AM | Beyotime |
| 8 | Optimal cutting temperature compound | SAKURA Tissue-Tek |

**Table S2. Antibodies and Recombinant Proteins**

| Number | Antibodies and recombinant proteins | Source |
| --- | --- | --- |
| 1 | anti-mouse CD47 mAb | Bio X Cell |
| 2 | CD45-PerCP-Vio700 | Thermo Fisher Scientific |
| 3 | CD11b-PE-Vio615 | Thermo Fisher Scientific |
| 4 | F4/80-APC | Thermo Fisher Scientific |
| 5 | CD80-FITC | Thermo Fisher Scientific |
| 6 | CD206-PE | Thermo Fisher Scientific |
| 7 | Mouse IL-4 Recombinant Protein | PeproTech |
| 8 | Mouse IL-13 Recombinant Protein | PeproTech |
| 9 | Mouse M-CSF Recombinant Protein | PeproTech |

**Table S3. Chemicals and Peptides**

| Number | Chemicals and peptides | | Source |
| --- | --- | --- | --- |
| 1 | 1, 8-naphthalimide | Aladdin | |
| 2 | N,N-Dimethylformamide（DMF） | Aladdin | |
| 3 | Iodoethane | Aladdin | |
| 4 | Methylmagnesium chloride | Aladdin | |
| 5 | dimethyl sulfoxide (DMSO) | Sigma-Aldrich | |
| 6 | DSPE-PEG_2000_ | Nanocs | |
| 7 | DSPE-PEG_2000_-MAL | Nanocs | |
| 8 | M2pep | Sangon Biotech | |
| 9 | IPI549 | MedChemExpress | |

**2. Supplementary Methods**

**Synthesis of intermediate**

A dried 500 mL three-necked flask was charged with 1,8-naphthalimide (8.0 g, 47 mmol, 1.0 eq) dissolved in 40 mL anhydrous DMF under ultrasonication. Under nitrogen protection and ice-bath cooling, a suspension of NaH (1.7 g, 71 mmol, 1.5 eq) in DMF was added dropwise. After stirring in the ice bath for 30 min, iodoethane (6 mL, 72 mmol, 1.5 eq) was added dropwise as the alkylating agent. The mixture was stirred at room temperature under nitrogen for 6 h. Upon completion, the reaction mixture was poured into water in batches to prevent vigorous reaction of NaH. The product was extracted with ethyl acetate (3×), concentrated by rotary evaporation, and dried to afford compound 1 as a yellow viscous liquid (8.3 g, 90% yield).

For compound 2, compound 1 (1.4 g, 7.1 mmol, 1.0 eq) was added to a 100 mL Schlenk flask with 20 mL anhydrous tetrahydrofuran (THF). The flask was sealed and purged with nitrogen. Under ice-bath cooling, methylmagnesium chloride (1.0 mL, 13.3 mmol, 2.0 eq) was added dropwise with vigorous stirring. After the solution turned dark green, stirring continued at room temperature for 10 min, followed by heating to 60 °C for 2 h. After cooling to room temperature, 8 mL of 3 mol/L HCl was added to quench the reaction. THF was removed by rotary evaporation, and a 0.3 g/mL aqueous KI solution was added dropwise to the residue with stirring, yielding an orange-red precipitate. The solid was collected by filtration and dried to afford compound 2 (1.65 g, 72% yield).

ET1080 was synthesized by adding compound 2 (800 mg, 2.5 mmol, 2.1 eq) and N-[(3-(anilinomethylene)-2-chloro-1-cyclohexen-1-yl)methylene]aniline hydrochloride (430 mg, 1.2 mmol, 1.0 eq) to a 50 mL Schlenk flask. The solids were dissolved in 3 mL acetic acid by ultrasonication, followed by addition of acetic anhydride (0.5 mL) and triethylamine (0.5 mL), turning the solution dark green. Under nitrogen, the mixture was heated in an oil bath at 60 °C for 1 h. After cooling to room temperature, 20 mL ethyl acetate was added to precipitate the product. The solid was collected by filtration and dried to afford ET1080 as a dark purple solid (457 mg, 73% yield).

**Synthesis of ETNO**

ETNO was synthesized by adding ET1080 (266 mg, 0.5 mmol, 1.0 eq) and p-anisidine (307 mg, 2.5 mmol, 5.0 eq) to a dried 250 mL round-bottom flask. The mixture was dissolved in 5 mL anhydrous DMF under ultrasonication and stirred at room temperature under nitrogen for 12 h. The crude product was purified by silica gel column chromatography using a gradient elution of dichloromethane/methanol (10:1). Concentration and drying afforded ETNO as a dark green solid (263 mg, 71% yield). The product was characterized by HR-MS and NMR spectroscopy.

**Theoretical calculations**

The density functional theory (DFT) calculations were performed using Gaussian 09W software to determine the highest occupied molecular orbital (HOMO) and lowest unoccupied molecular orbital (LUMO) energy levels of ET1080, ETNO, and ETNO-t (ETNO treated with NO). The geometry optimization was conducted with the PBE1PBE hybrid functional and DEF2-TZVP basis set. This computational approach allowed precise characterization of intramolecular charge transfer (ICT) effects and electronic transitions during NO-responsive structural modifications.

**Characterization of ETNO**

The molecular weight of ETNO is 741.22. One milligram of ETNO dry powder was weighed and dissolved in anhydrous ethanol to prepare a 400 μM stock solution. 100 μL of the stock solution was transferred to five centrifuge tubes using a pipette. The solvent was then gently blown dry with nitrogen gas. Subsequently, 1 mL of ultrapure water, acetonitrile (ACN), ethanol, DMSO, acetone, and dichloromethane (DCM) was added to each centrifuge tube, respectively, and the mixtures were sonicated for 2 min to ensure stabilization. The absorption spectra of the solutions were measured in the range of 400-1100 nm using a UV-Vis spectrophotometer, and the fluorescence spectra were recorded in the range of 900-1500 nm using a near-infrared fluorescence spectrometer.

**Relative determination of fluorescence quantum yield (QY)**

The relative QY of ETNO and ETNO-t was determined using IR-26 (QY = 0.05% in 1,2-dichloroethane) as the reference standard according to established protocols. Gradient concentrations (10 μM, 7.5 μM, 5 μM, 2.5 μM) of IR-26 were prepared by serial dilution from a 100 μM stock solution in 1,2-dichloroethane. Absorption spectra (400-1100 nm) and fluorescence spectra (900-1500 nm) were acquired using a UV-Vis-NIR spectrophotometer and NIR fluorescence spectrometer, respectively. The integrated fluorescence intensity (900-1300 nm for ETNO/ETNO-t; 1000-1400 nm for IR-26) was plotted against the corresponding absorbance at 887 nm (ETNO) or 940 nm (IR-26) to generate linear calibration curves. The slopes of these curves (slope ref for IR-26; slope sample for ETNO/ETNO-t) were substituted into the modified Strickler-Berg equation:

QY_sample_=QY_ref_×$\frac{{Slope}_{sample}}{{Slope}_{ref}}$×$\frac{n_{sample}^{2}}{n_{ref}^{2}}$

Here, n_sample_ and n_ref_ respectively represent the refractive indices of the sample solution and the reference solution. Three independent measurements were conducted to ensure reproducibility.

**Calculation of the photothermal conversion efficiency**

The absorbance of I/E@M2pep solution (40 μM, 1 mL) was measured using a quartz cuvette. A continuous-wave laser (808 nm, 1 W cm⁻²) was employed as the excitation source, and the laser power was monitored and controlled with a power meter. The cuvette containing the probe solution was irradiated while the temperature was recorded every 30 s using a thermocouple. The irradiation was continued until the solution reached a steady-state temperature, after which the laser was turned off, and the cooling process was recorded until the temperature returned to ambient conditions. The photothermal conversion efficiency (η) was calculated according to the following equation:

𝜂 = $\frac{hs\left( {\Delta T}_{max}-{\Delta T}_{surr} \right)-Q_{dis}}{I\times(1-{10}^{-A\lambda}）}$

where η is the photothermal conversion efficiency, ℎ is the heat transfer coefficient, 𝑆 is the surface area of the container, ∆𝑇_𝑚𝑎𝑥_ is the maximum temperature increase relative to the ambient temperature, ∆𝑇_surr_​ denotes the ambient temperature, 𝐼 is the incident laser power, 𝐴_λ_ is the absorbance of the probe solution at 808 nm (or 1064 nm), and 𝑄_𝑑𝑖𝑠_ represents the heat dissipated by the solvent and cuvette. The heat transfer coefficient was obtained using:

ℎ𝑆 = $\frac{mc_{water}}{t}$×lnθ

with

θ = $\frac{T-T_{surr}}{\Delta T_{max}}$

where 𝑡 is the cooling time, 𝑚 is the mass of the solution, 𝐶_𝑤𝑎𝑡𝑒𝑟_​ is the specific heat capacity of water, 𝑇_surr_​ denotes the ambient temperature, and ∆𝑇_𝑚𝑎𝑥_ is the maximum temperature increase relative to the ambient temperature.

The laser setup for the photothermal stability evaluation was identical to that described above. Probe solutions before and after NO treatment were irradiated with an 808 nm laser for 15 min, followed by natural cooling for another 15 min. This heating–cooling cycle was repeated five times. The temperature variation versus time curves were recorded, and the photothermal stability was assessed based on the amplitude of the temperature changes.

3. Supplementary Figures


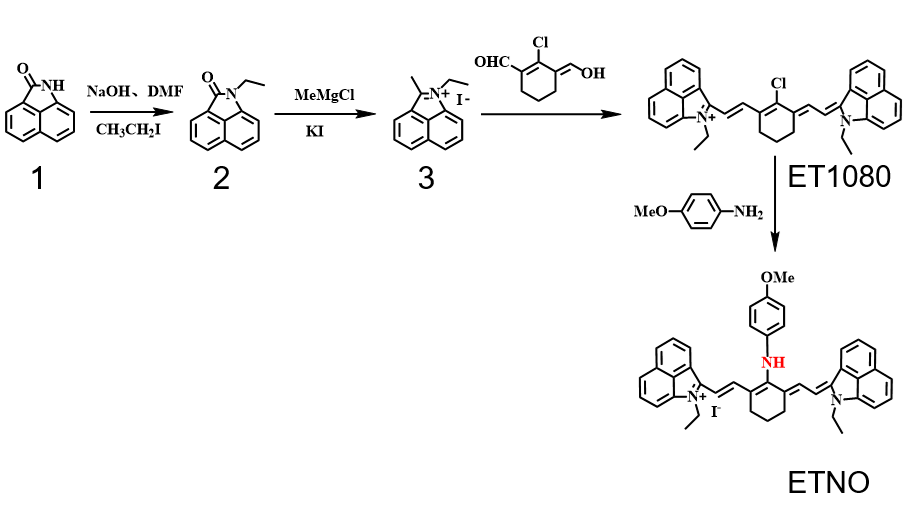
**Figure S1.** The synthesis pathway of ETNO.


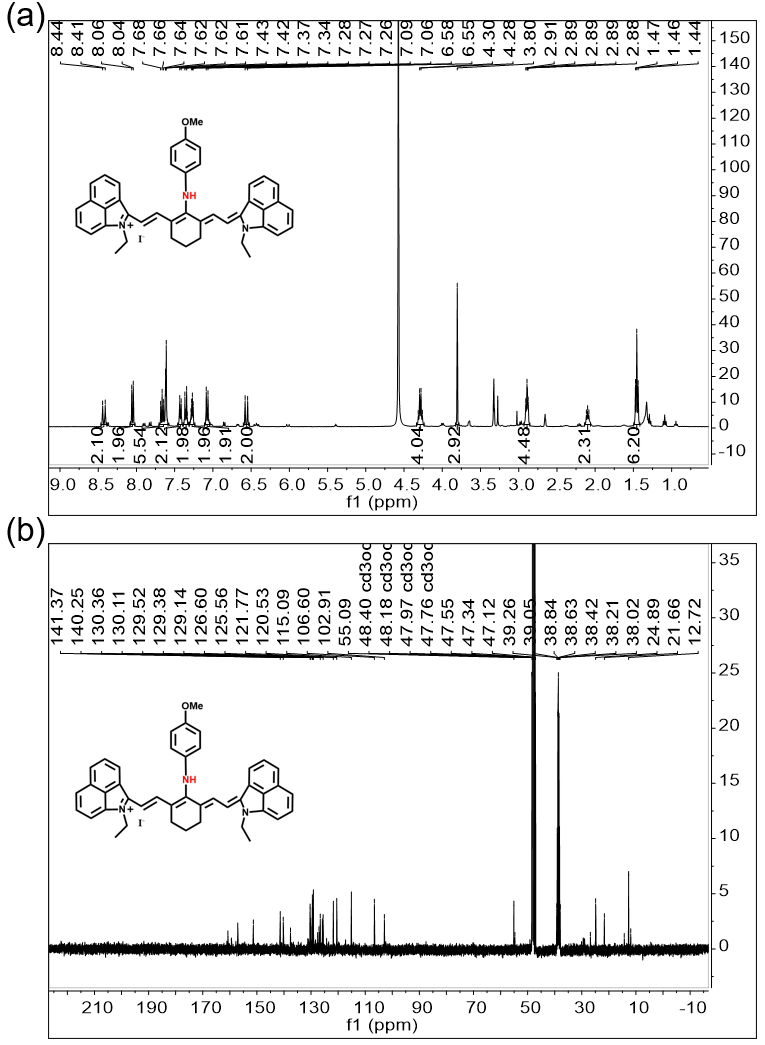


**Figure S2.** ^1^H NMR (a) and ^13^C NMR (b) of compound ETNO.


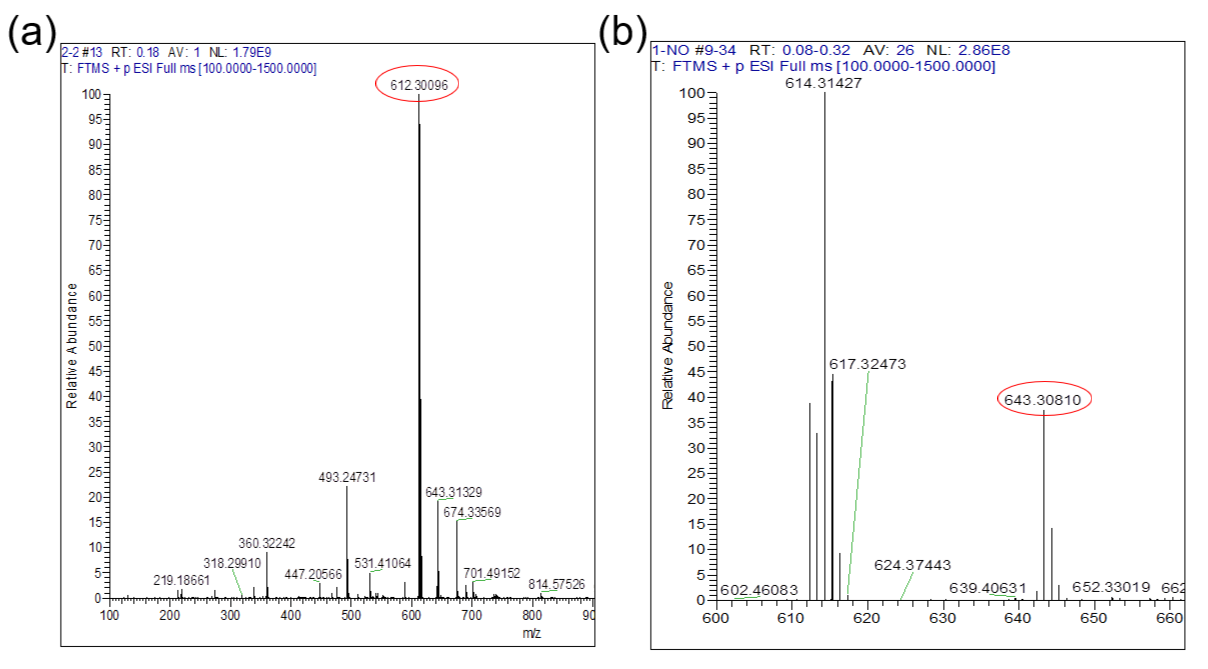


**Figure S3.** Mass spectra of compound ETNO (a) and ETNO-t (b).


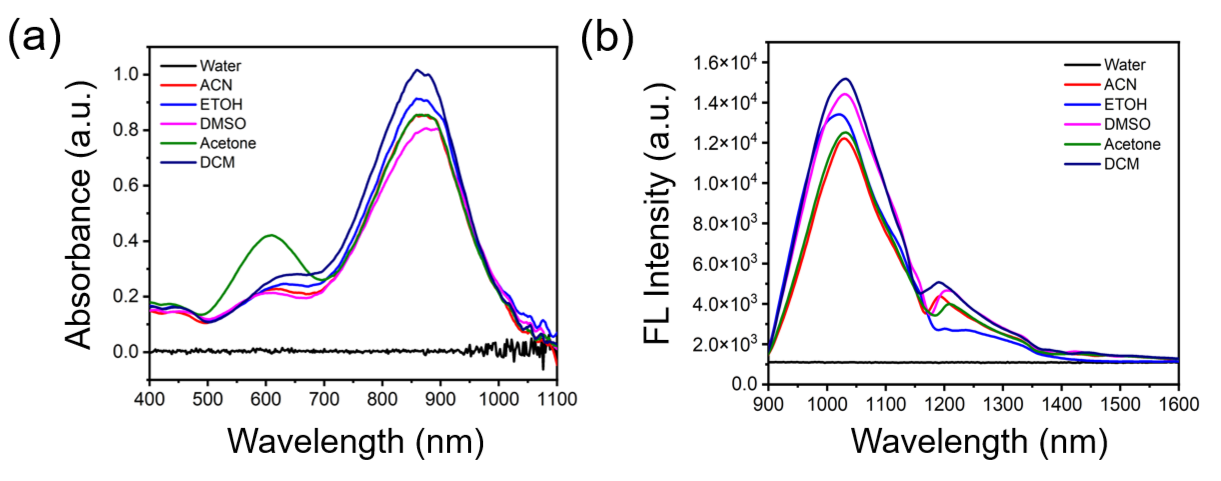


**Figure S4.** Absorption (a) and emission (b) spectra of ETNO in different solvents.


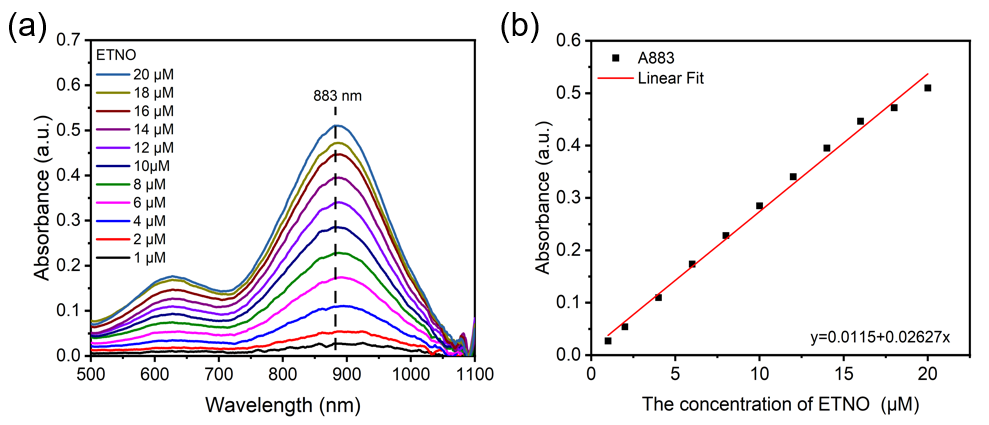


**Figure S5.** Gradient concentration absorption spectra ( a ) and measured molar extinction coefficient ( b ) of ETNO.


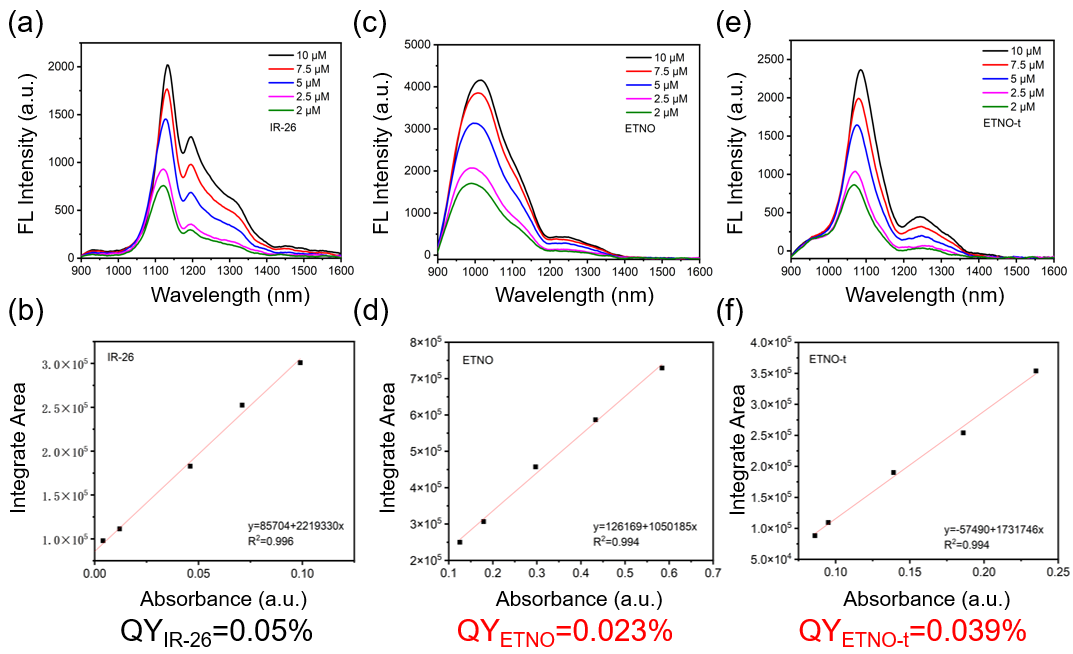
**Figure S6.** Calculation of relative quantum yield. (a) The fluorescence spectra of IR-26 and integral area-absorbance ratio diagram (b). (c) The fluorescence spectra of ETNO and integral area-absorbance ratio diagram (d). (e) The fluorescence spectra of ETNO-t and integral area-absorbance ratio diagram (f).

**
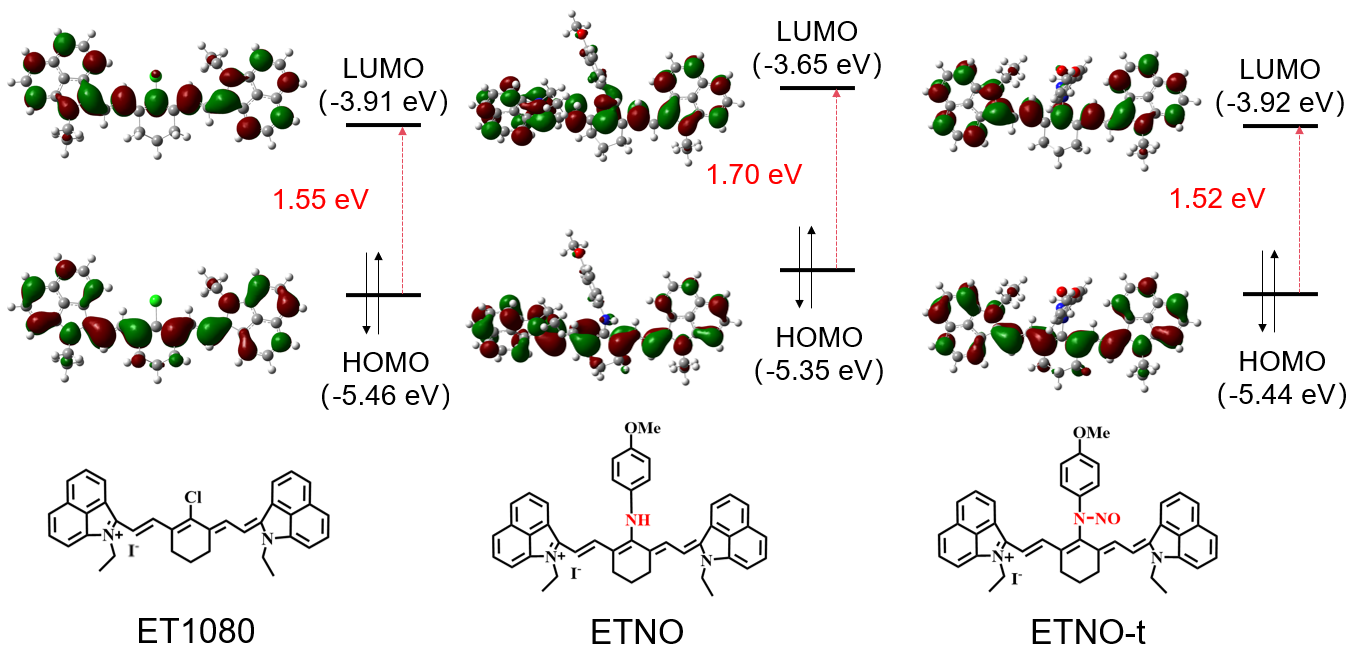
Figure S7.** DFT calculated HOMO and LUMO energy levels and orbital distributions of ET1080, ETNO and ETNO-t.


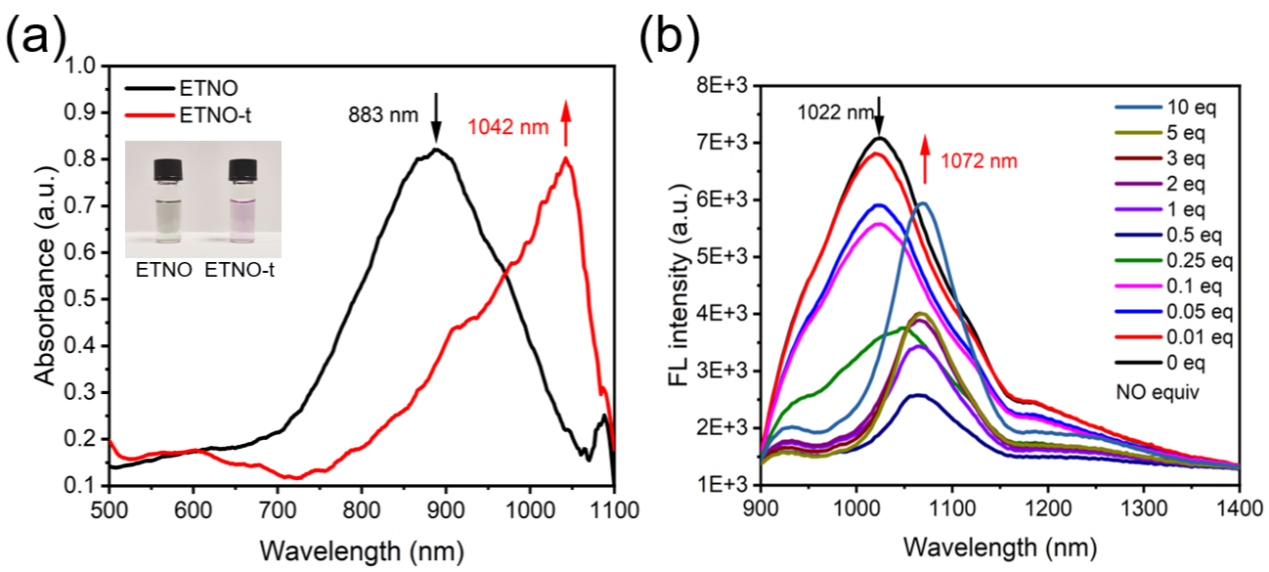
**Figure S8.** (a) Absorption spectra and photographs of ETNO before and after treatment with NO. (b) Fluorescence spectra of ETNO treatment with different concentrations NO.


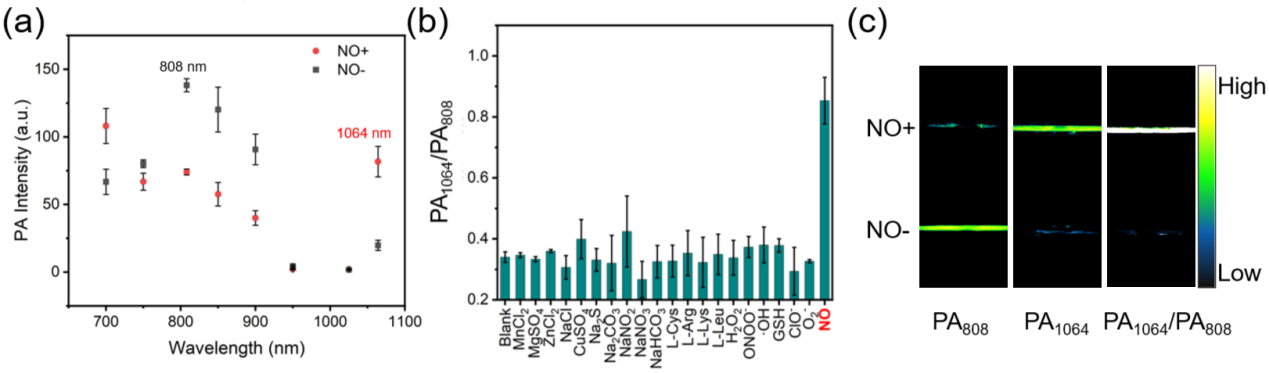
**Figure S9.** (a) PA intensity of ETNO under different excitation wavelengths (n=3). The error bars indicate SD. (b) The NO ratio PA selectivity of ETNO (n=3). The error bars indicate SD. (c) PA images of ETNO before and after treatment with NO.


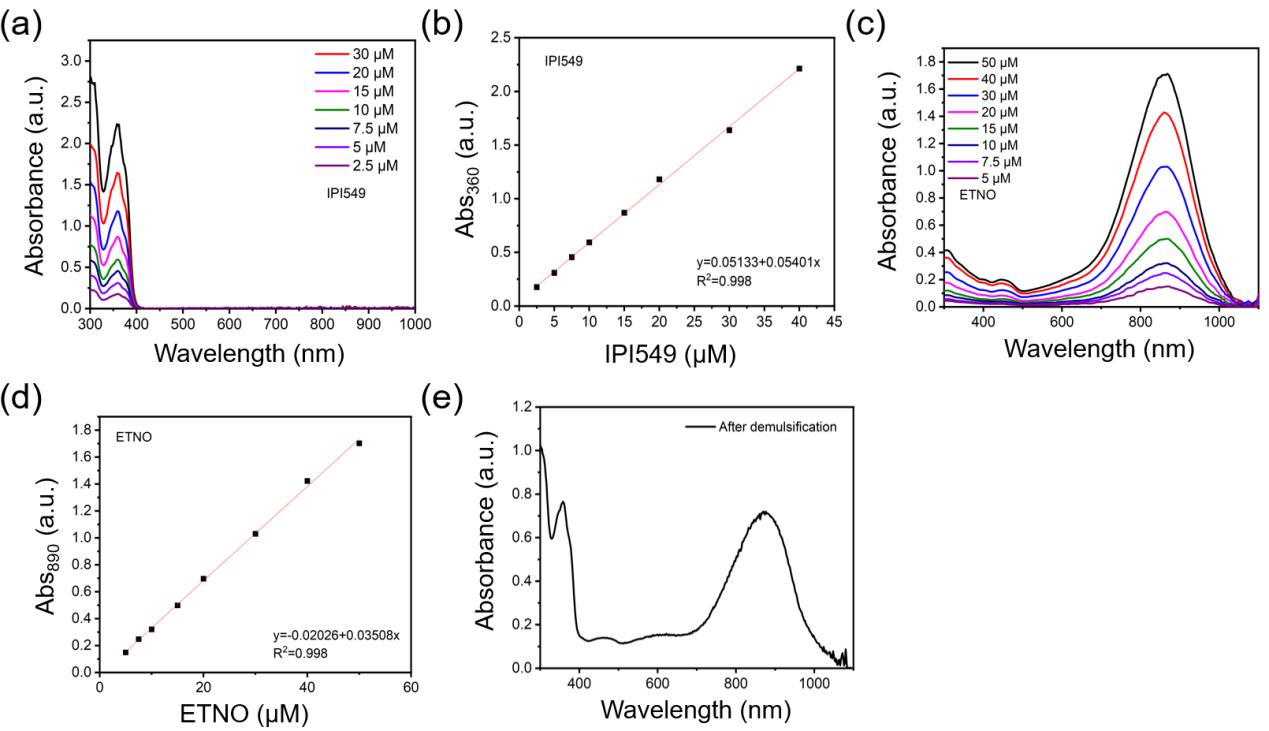


**Figure S10.** Gradient absorption plot (a), linear correlation plot (b) of IPI549, gradient absorption plot (c), linear correlation plot (d) of ETNO and micelle encapsulation efficiency determination plot (e).


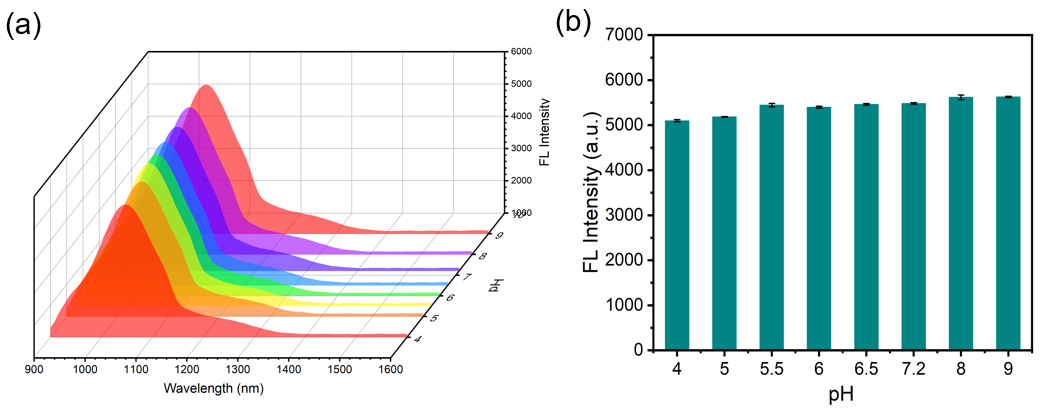
**Figure S11.** Fluorescence spectra (a) and stability test (b) of I/E@M2pep at different pH values. The error bars indicate SD.


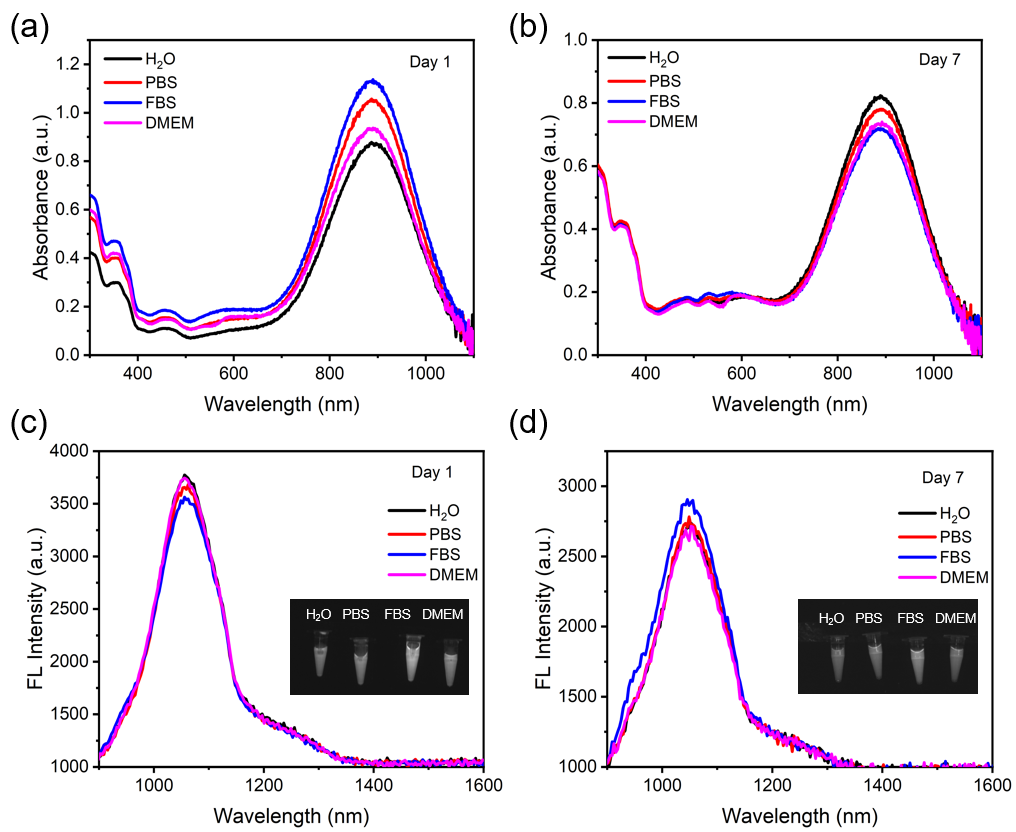
**Figure S12.** The stability test of I/E@M2pep in different solvents. The absorption spectra and fluorescence spectra of the first (a, c) and seventh days (b, d).


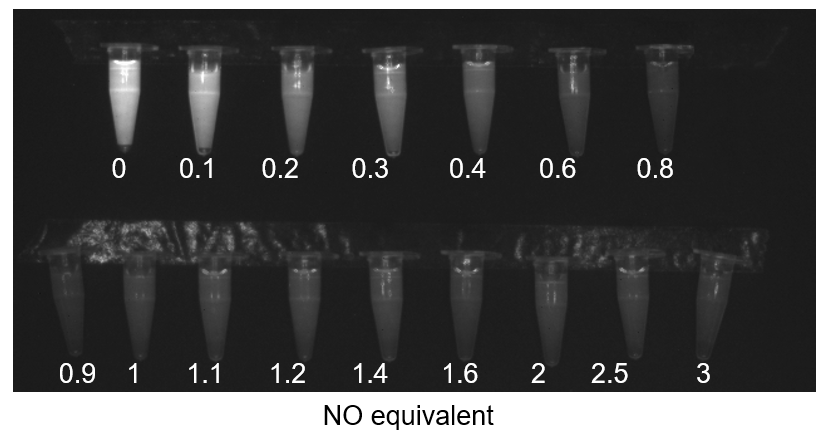
 **Figure S13.** FL images of I/E@M2pep before and after incubation with different concentrations of NO.


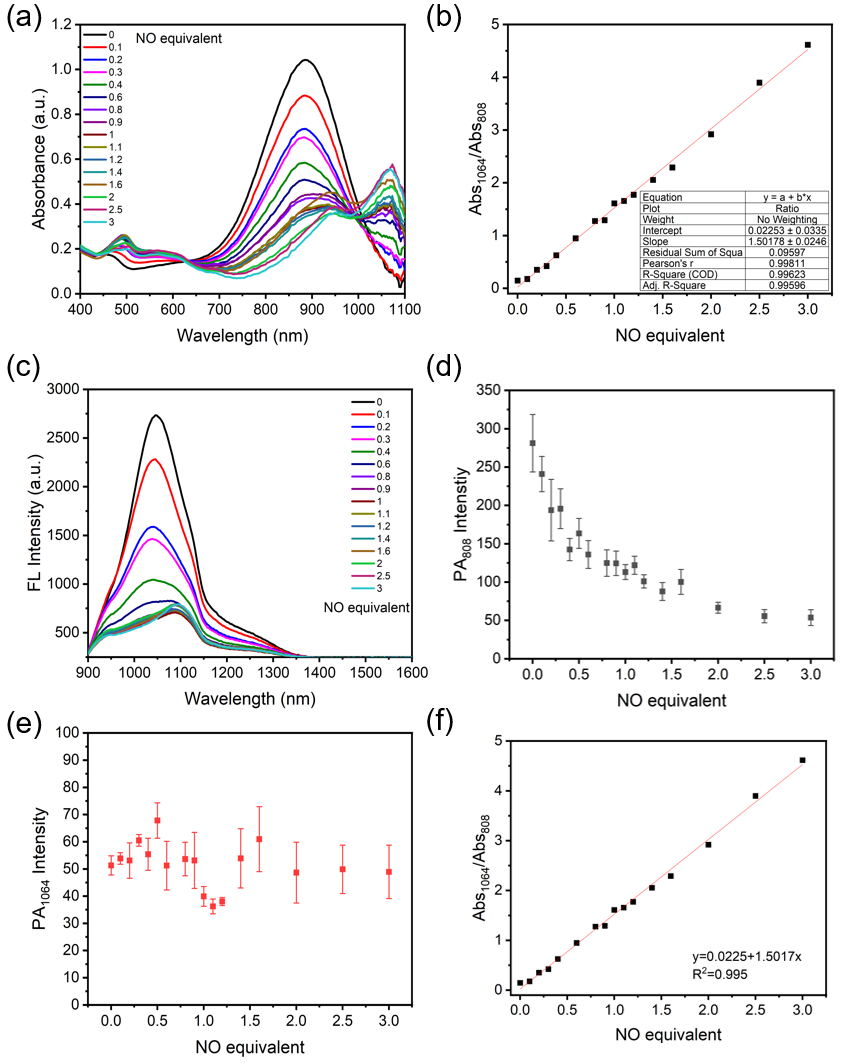
**Figure S14.** The absorption spectra (a) and ratio absorption spectra (b) of I/E@M2pep before and after incubation with different concentrations of NO.


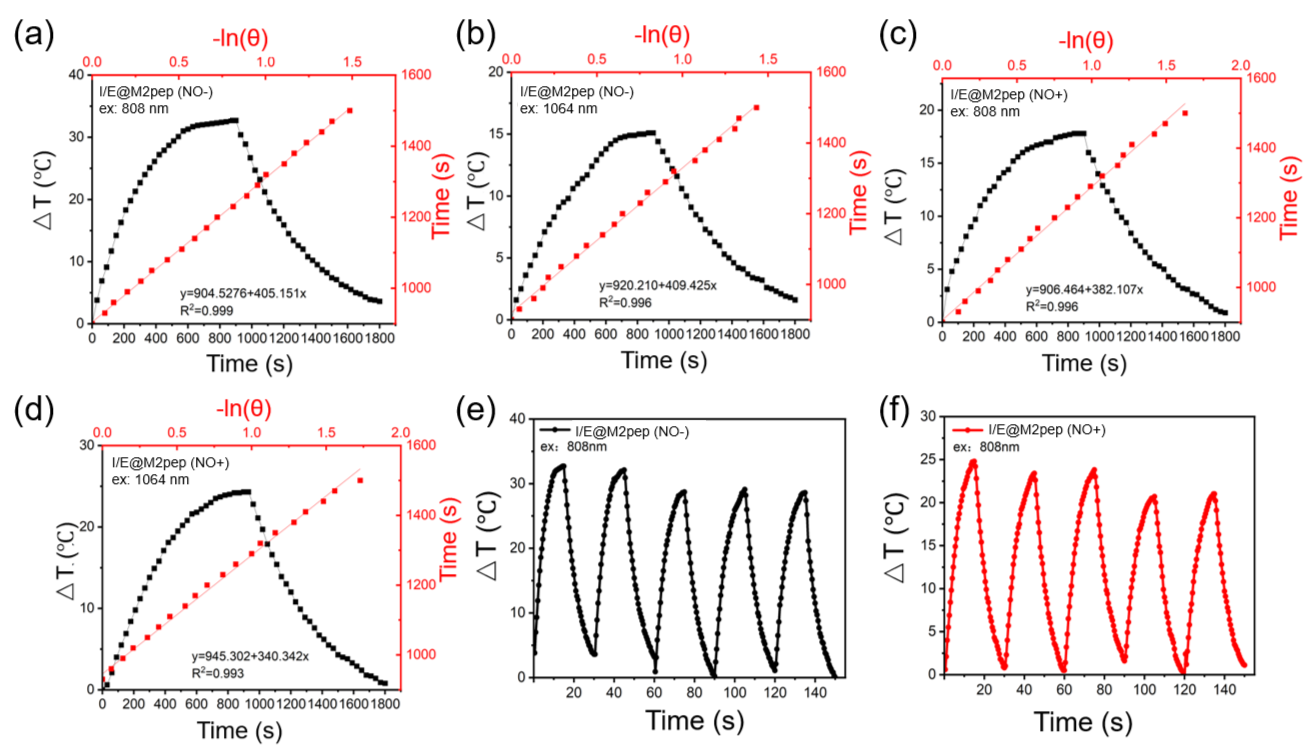
**Figure S15.** Photothermal conversion efficiency (a-d) and photothermal stability test (e, f) of I/E@M2pep before and after NO treatment.


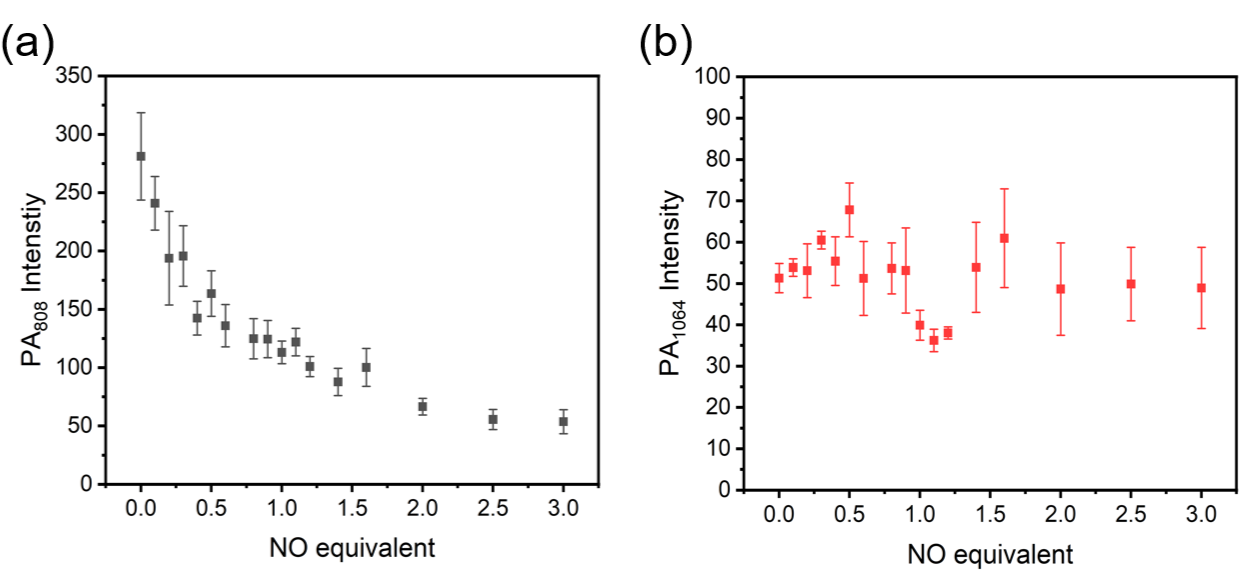
 **Figure S16.** The PA_808 nm_ photoacoustic signal (a) (n=3) and PA_1064 nm_ photoacoustic signal (b) (n=3) of I/E@M2pep before and after incubation with different concentrations of NO. The error bars indicate SD.


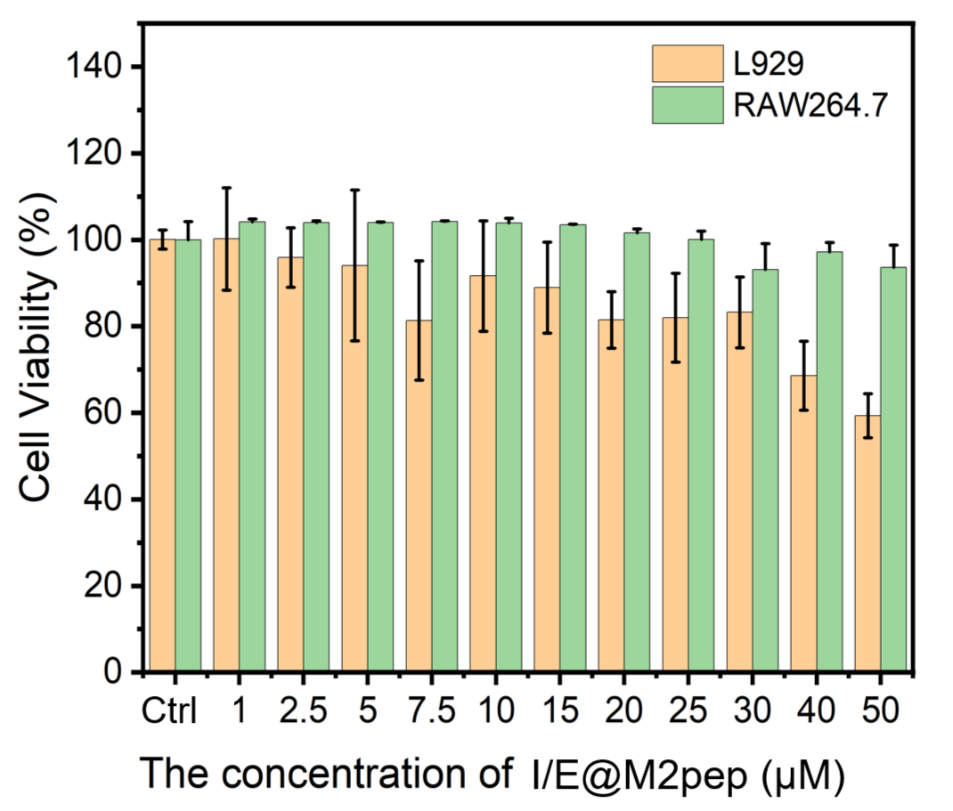


**Figure S17.** L929 and RAW264.7 cells were used to test the toxicity of the nanoinducer (n=3). The error bars indicate SD.


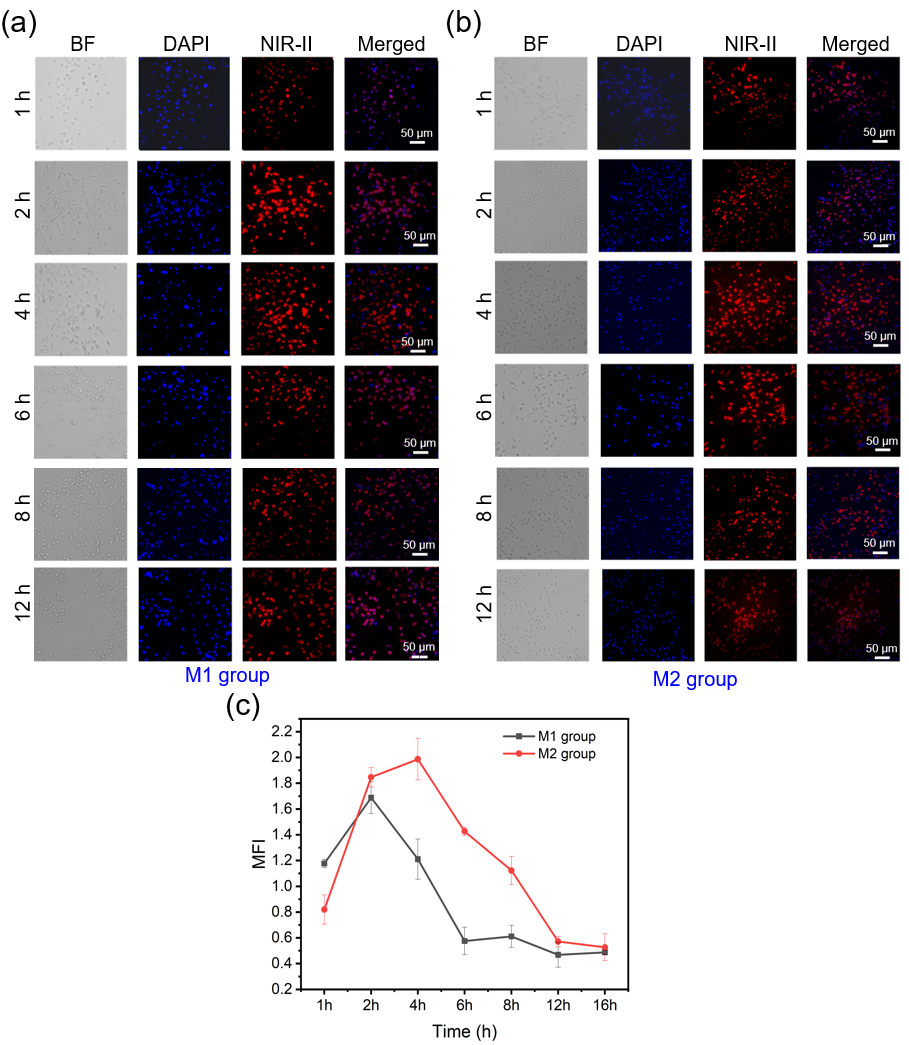


**Figure S18.** Fluorescence images (a, b) and intensity analysis (c) (n=3) of cellular uptake I/E@M2pep in M1 (a) and M2 (b) macrophages. The error bars indicate SD.


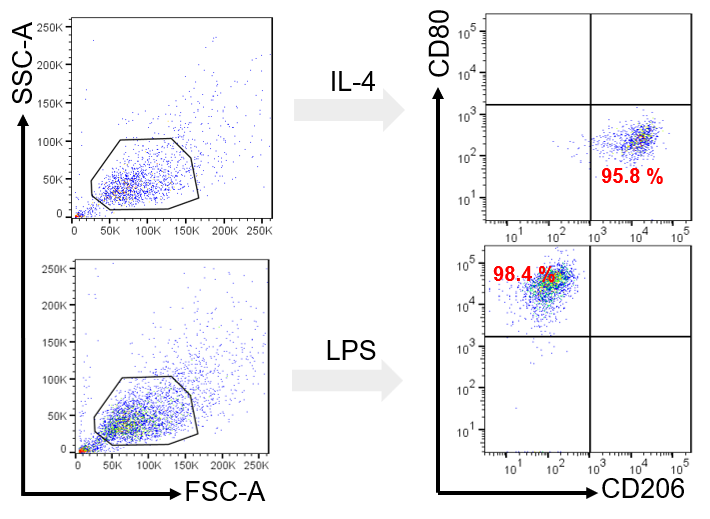
**Figure S19.** M1 and M2 macrophages were sorted by flow cytometry.


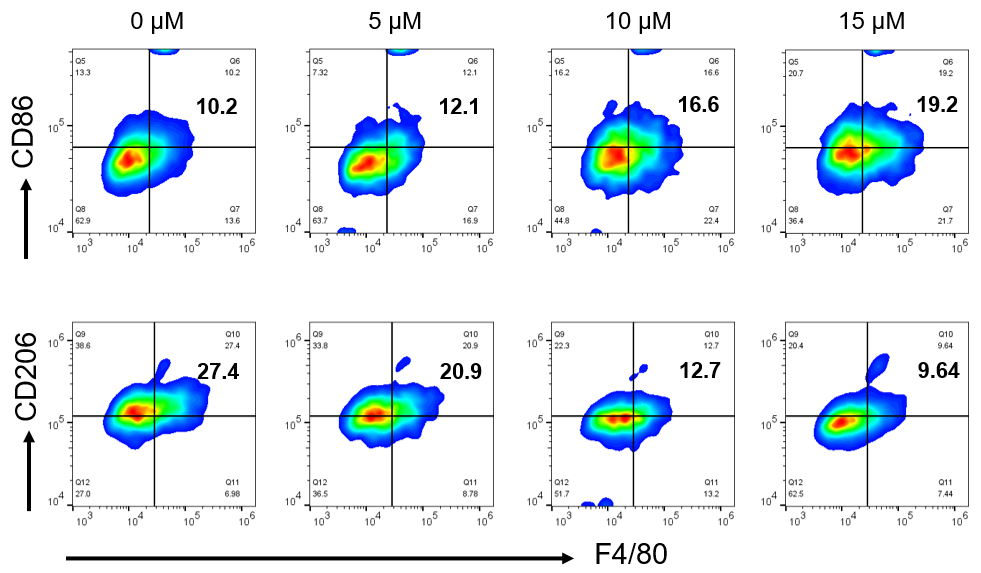


**Figure S20.** Flow cytometry analysis of M2 macrophages and dynamic changes of two macrophage subsets after treatment with IPI549.

**
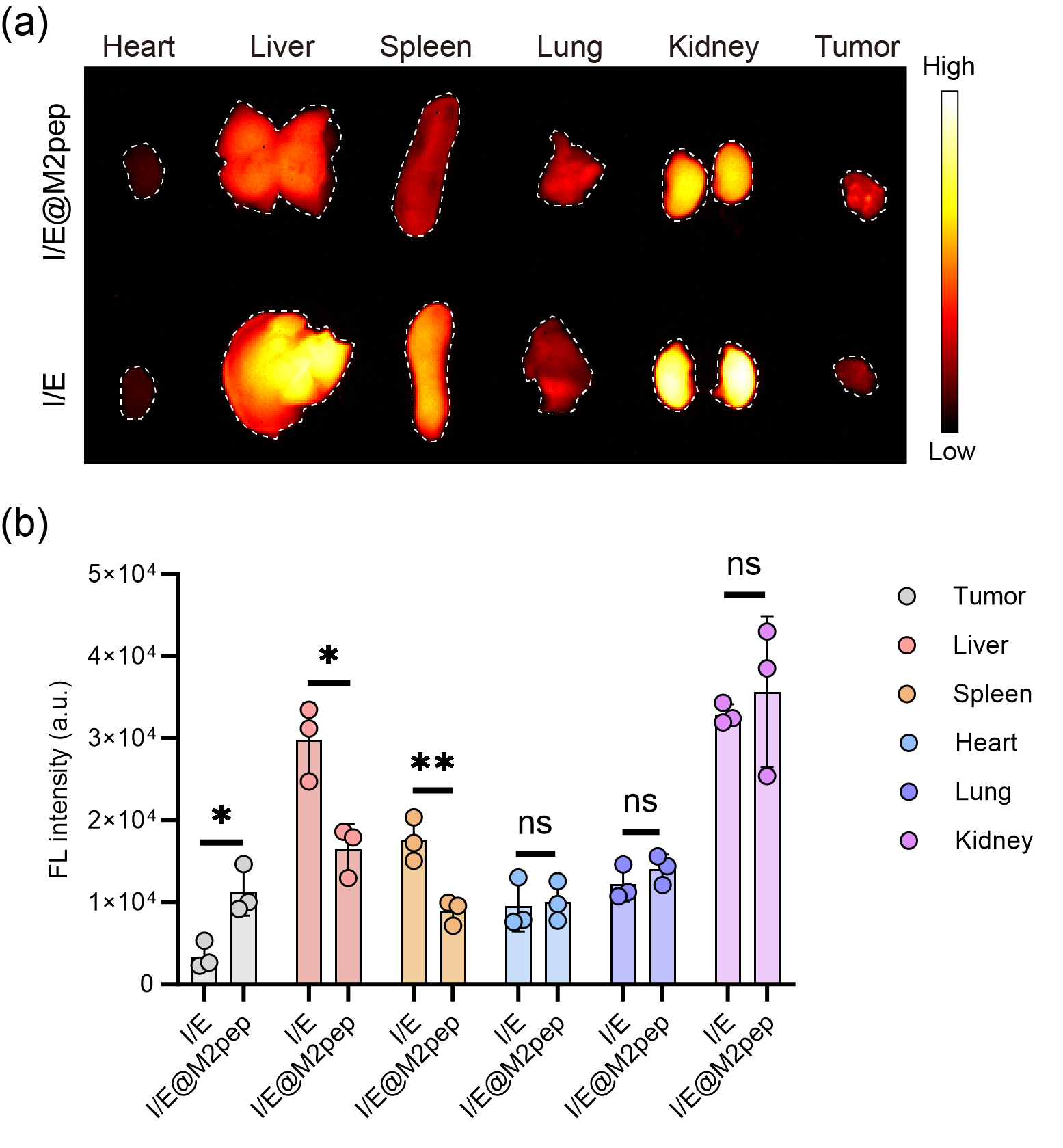
**
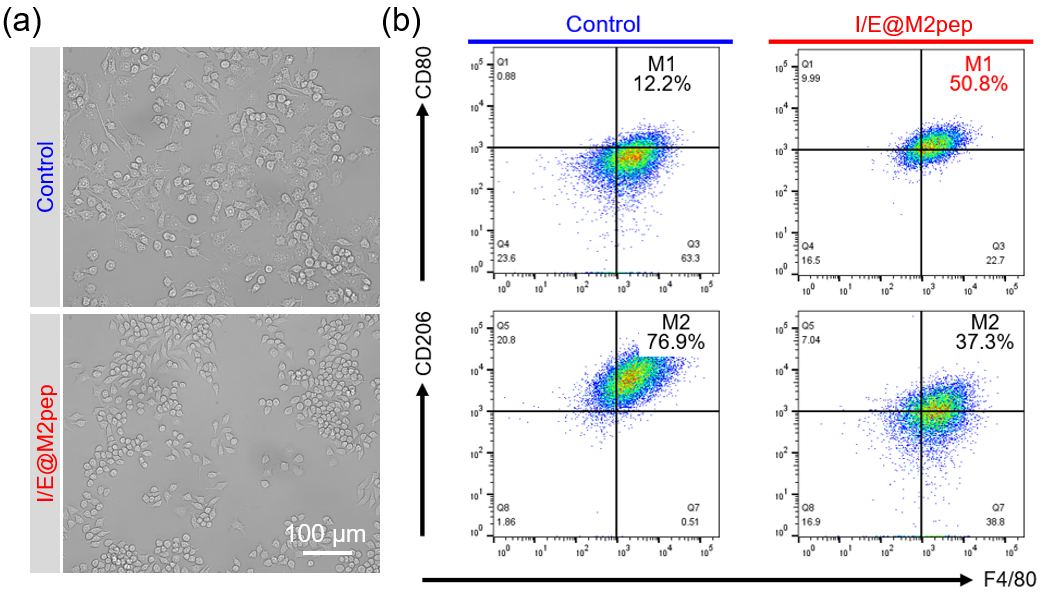
**Figure S21.** Morphological (a) and phenotypic (b) changes of M2 RAW264.7 cells treated with I/E@M2pep.

**Figure S22. Fluorescence images of I/E@M2pep and I/E in tumor and major organs.** (a) NIR-II Fluorescence images of I/E@M2pep and I/E in tumor and major organs. (b) Fluorescence quantitative analysis of in A (n=3). Statistical significance: All data are presented as the mean ± SD. **p* < 0.05, ***p* < 0.01, ****p* < 0.001, *****p* < 0.0001, as determined by one-way ANOVA.


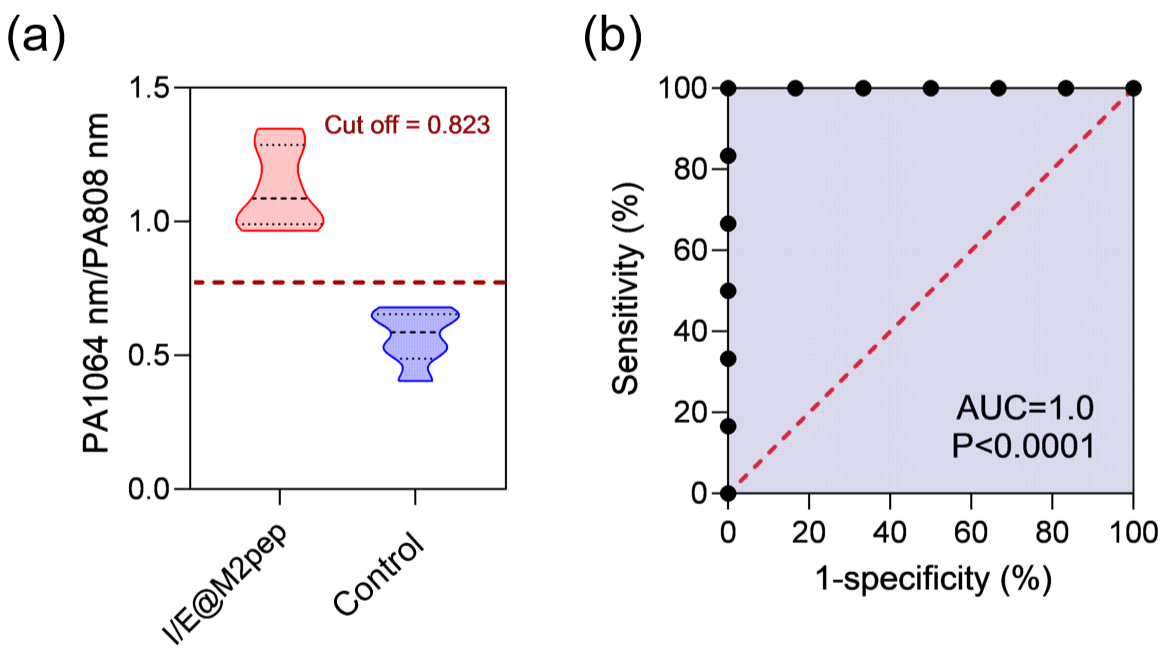
**Figure S23. The ratiometric PA strategy for accurately evaluating the repolarization effect of macrophages.** (a) The ratiometric PA signal in both groups. (b) ROC curve for evaluating the repolarization effect of macrophages. When the cut-off value of the ratio is set to 0.823, the AUC is 1.0.


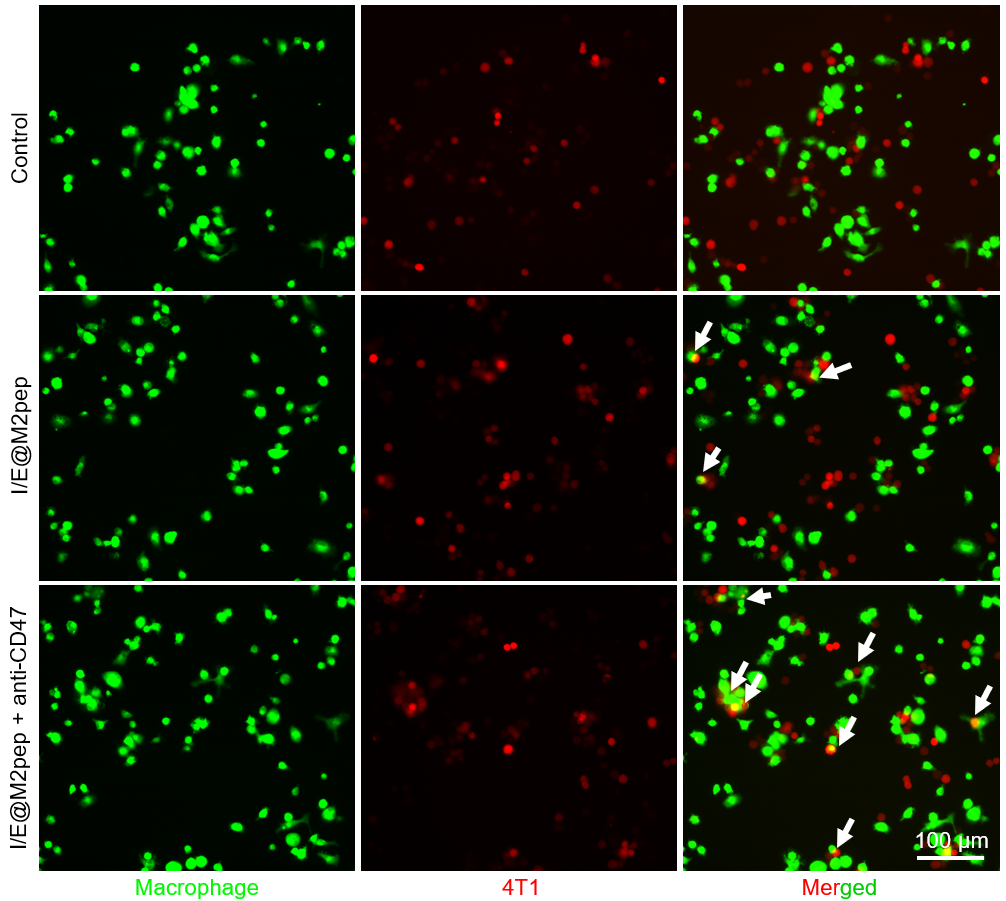


**Figure S24.** Macrophages (BMDM) phagocytosing tumor cells following treatment with I/E@M2pep with or without CD47 mAb. Arrows point to phagocytosed tumor cells.


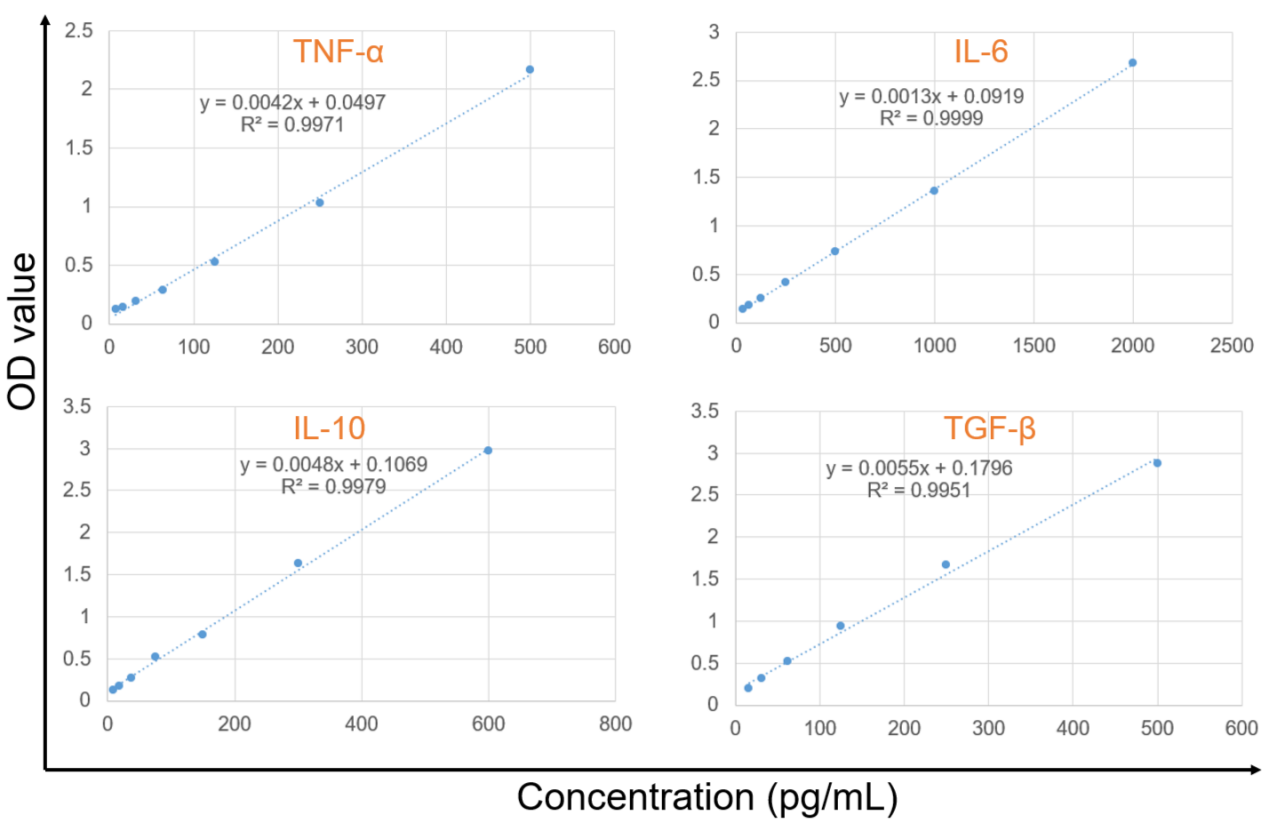


**Figure S25.** The standard curve of TNF-α, IL-6, IL-10, TGF-β drawn by ELISA.


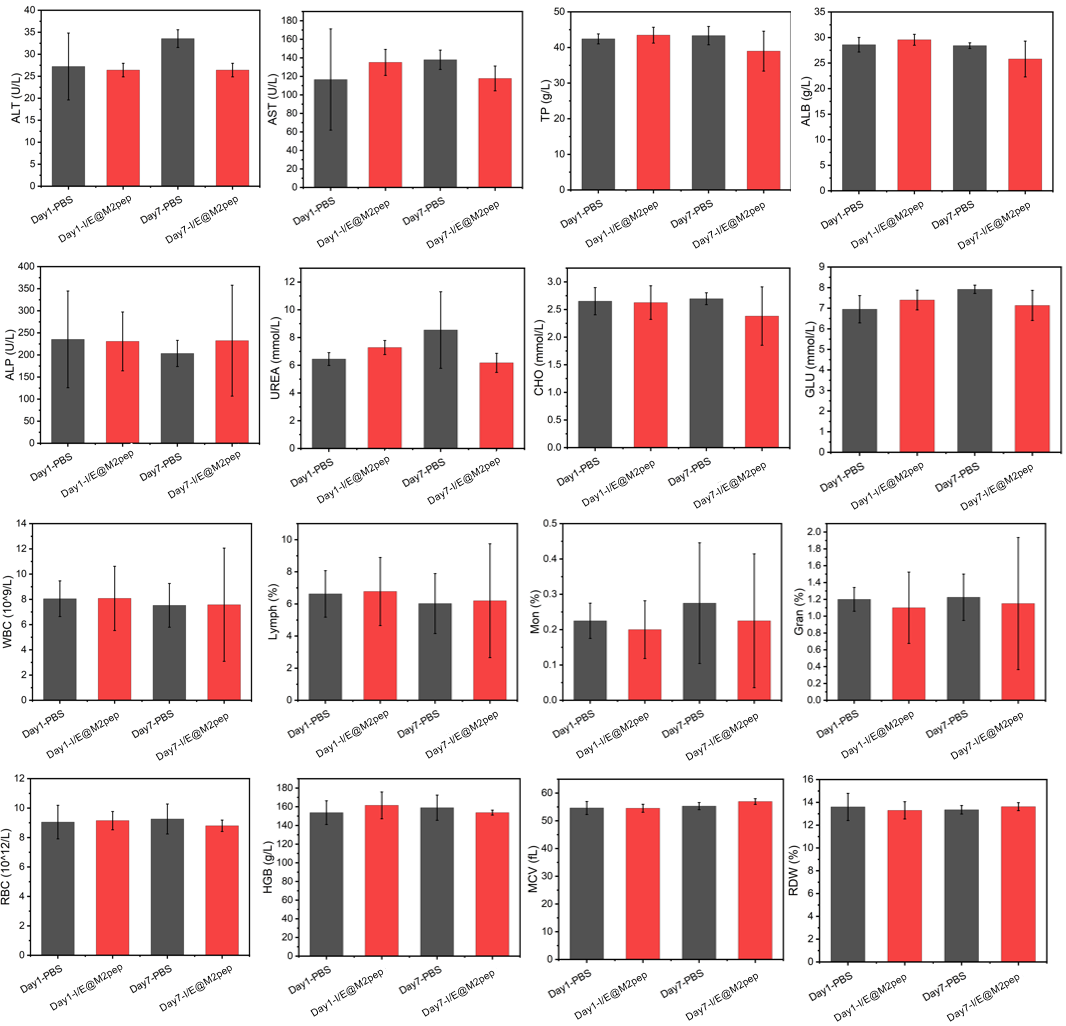


**Figure S26.** *In vivo* safety evaluation of the combined therapy by blood routine examination and blood biochemical examination at days 1 and day 7 after different administrations (n=3). The error bars indicate SD.


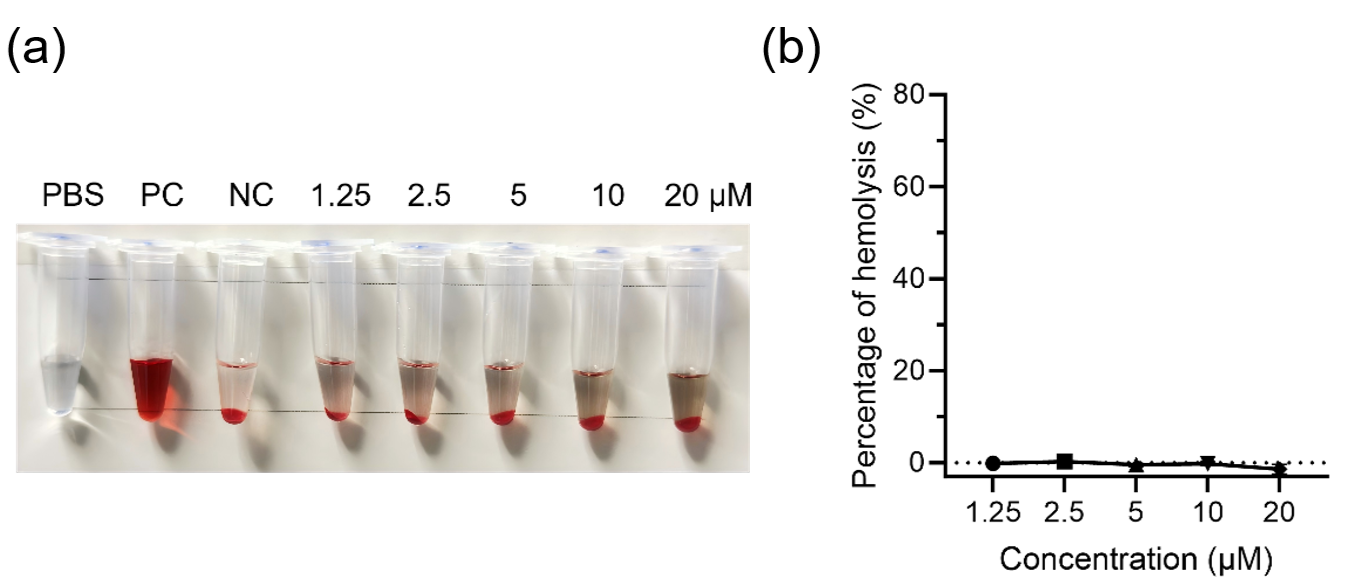


**Figure S27. Hemolytic and cell toxicity of I/E@M2pep.** (A) Hemolytic toxicity of I/E@M2pep at different concentrations. (B) Hemolytic toxicity quantitative analysis of I/E@M2pep.


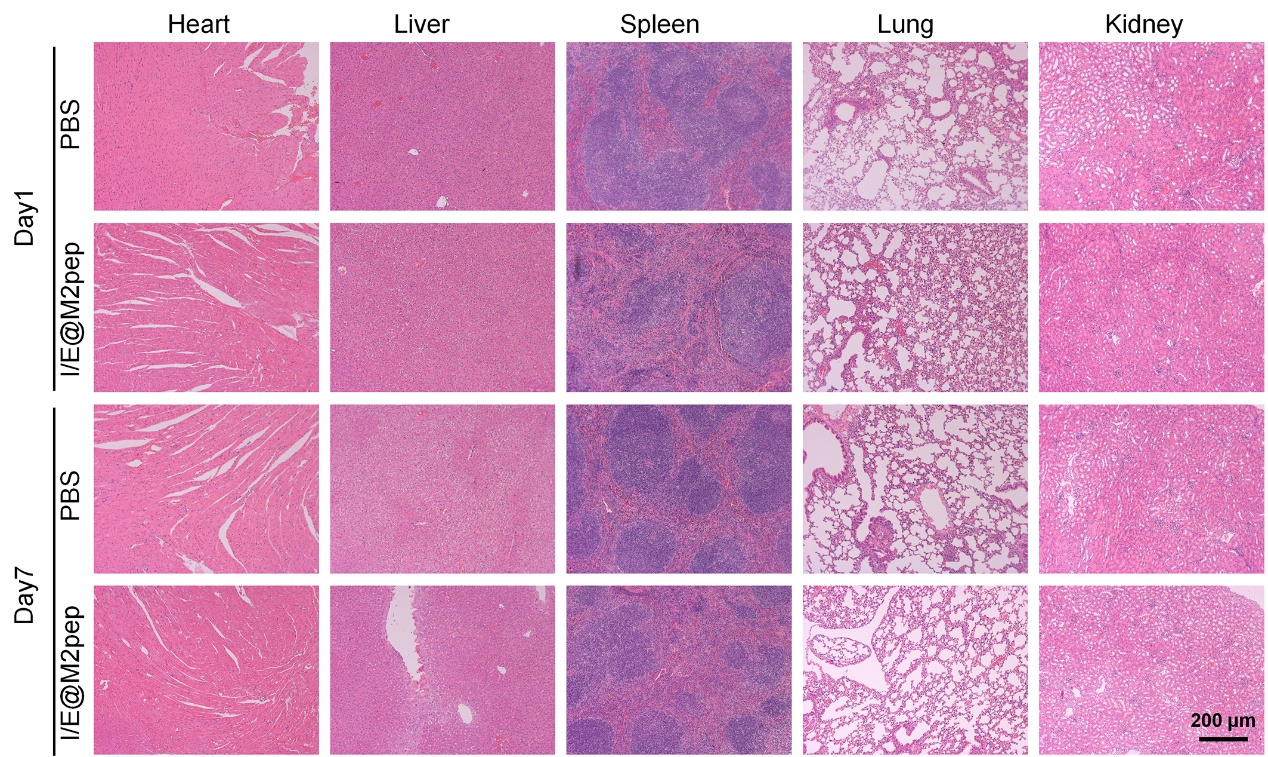
**Figure S28.** The H&E staining analysis of mice mainly organs (heart, liver, spleen, lung, kidney) at days 1 and day 7 after different administrations.
